# Low glycaemic index diet in pregnancy and child asthma: follow-up of the ROLO trial

**DOI:** 10.1017/S0007114524001612

**Published:** 2026-03-14

**Authors:** Sophie Callanan, Mohammad Talaei, Anna Delahunt, Seif O. Shaheen, Fionnuala M. McAuliffe

**Affiliations:** 1 UCD Perinatal Research Centre, School of Medicine, University College Dublin, National Maternity Hospital, Dublin, Republic of Ireland; 2 Wolfson Institute of Population Health, Barts and The London School of Medicine and Dentistry, Queen Mary University of London, London, UK; 3 Allergy and Lung Health Unit, Melbourne School of Population and Global Health, The University of Melbourne, Melbourne, VIC, Australia

**Keywords:** Pregnancy, Asthma, Sugar, Maternal diet, Glycaemic index

## Abstract

Epidemiological evidence suggests that a higher intake of sugar during pregnancy is associated with a higher risk of childhood asthma and atopy. However, randomised trial evidence supporting such a link is lacking. This study aimed to examine whether a low glycaemic index (GI) dietary intervention during pregnancy decreases the risk of childhood asthma and eczema. This is a secondary analysis of 514 children from the ROLO trial. Healthy women were randomised to receive an intervention of low GI dietary advice or routine care from early pregnancy. Mothers reported current doctor-diagnosed eczema in their children at 2 years (*n* 271) and current doctor-diagnosed asthma and eczema in their children at 5 (*n* 357) and 9–11 years (*n* 391) of age. Multivariable logistic regression models were used test the effect of the intervention on child outcomes overall and stratified by maternal education. There was a suggestion of a reduction in asthma at 5 years of age in children whose mothers received the low GI dietary intervention during pregnancy compared with usual care (adjusted OR 0·46 (95 % CI 0·19, 1·09); *P* = 0·08). In stratified adjusted analyses, the intervention was associated with a reduced risk of asthma at 5 years of age in children born to mothers with incomplete tertiary level education but not in those with complete tertiary level education (OR 0·14 (95 % CI 0·02, 0·69); *P* = 0·010 and OR 1·03 (95 % CI 0·34, 3·13); *P* = 0·94, respectively). A low GI diet in pregnancy may reduce the risk of developing asthma in childhood, particularly amongst children born to mothers with lower educational attainment.

Atopic diseases, including asthma and eczema, represent a substantial public health problem in children and adolescents globally; asthma is the commonest chronic disorder of childhood^(1)^. Evidence suggests that the origins of childhood asthma may lie in utero^(2)^, and several components of the maternal diet during pregnancy have been investigated in relation to atopic outcomes in children including intakes of vitamins and minerals, specific food groups (i.e. meat, fish, dairy, fruits and vegetables) and the Mediterranean diet pattern^(3,4)^. However, observational evidence is conflicting, and a recent European meta-analysis found no relation between maternal diet quality and childhood asthma^(5)^. Recent trials of vitamin D and fish oil supplementation in pregnancy, however, have shown promise for the prevention of preschool wheeze and early childhood asthma^(6)^, respectively, but their long-term effects are uncertain^(7,8)^.

Also of interest, two birth cohort studies have implicated a higher intake of sugar in pregnancy in the aetiology of childhood asthma and atopy. In 2017, in the Avon Longitudinal Study of Parents and Children (ALSPAC), a higher maternal intake of free sugar during pregnancy was associated with an increased risk of atopy (measured by aeroallergen skin prick tests) and atopic asthma in children at 7 years of age, independently of early childhood sugar intake^(9)^. In 2018, in the Project Viva cohort in the USA, higher intakes of sugar-sweetened beverage and fructose intake during pregnancy and early childhood were associated with an increased risk of asthma in 7-year-old children, irrespective of childhood adiposity^(10)^. Thus, different sources and forms of sugars may underlie these associations and the direct causal mechanisms remain unclear, partly due to varying definitions in the literature.

Despite these strong observational findings, the possibility of unmeasured or residual confounding cannot be ruled out, and randomised trial evidence supporting such a link is lacking. *De novo* trials of maternal dietary modification in pregnancy are time consuming and expensive, as children have to be followed up for at least 5 years in order to ascertain asthma development. However, another approach, which enables faster randomised evidence, is to follow-up previous trials, originally conducted with other outcomes in mind, and to follow-up the offspring to ascertain asthma through questionnaires or health record linkage^(11)^.

We therefore followed up children from the ROLO (Randomised cOntrol trial of a LOw glycaemic index diet in pregnancy to prevent macrosomia) trial to determine whether a randomised low glycaemic index (GI) dietary intervention during pregnancy is associated with lower risk of childhood asthma and eczema. We also aimed to assess observationally whether maternal intake of sugar during pregnancy is positively associated with asthma and eczema in childhood.

## Materials and methods

### Study design and subject selection

This is a secondary analysis of data on 514 children from follow-up of the ROLO trial, which was a randomised control trial of a low GI diet during pregnancy. The primary aim of the trial was to reduce the recurrence of macrosomia (birthweight >4 kg) in healthy secundigravida women who previously delivered an infant with macrosomia^(12)^. The trial was conducted in the National Maternity Hospital in Dublin, Ireland (2007–2011). A total of 800 women who met the eligibility criteria were randomly assigned to the intervention or control group. Detailed description of the trial criteria, methodology and outcomes has been previously reported^(12,13)^. Data were collected for 759 mother–child dyads at delivery. The original trial has developed into a longitudinal cohort, with subsequent follow-ups of mother–child dyads throughout childhood^(14,15)^.

### Ethical approval

The ROLO study and follow-ups were carried out in accordance with the Helsinki Declaration of 1975 as revised in 1983. Ethical approval for the primary ROLO trial, and the 2-year follow-up was obtained from the National Maternity Hospital Ethics Committee, Dublin, Ireland (GEN/279/12). Ethical approval for the 5- and 9–11-year follow-ups was obtained from the Ethics Committee (Medical Research) of Our Ladies Children’s Hospital Crumlin and University College Dublin, Office of Research Ethics Committee, Dublin, Ireland, respectively. The Current Controlled Trials registration number is ISRCTN54392969. Informed, written maternal consent was obtained prior to study participation, and verbal assent was obtained from the study child.

### Exposure assessment

#### Randomised trial of a low glycaemic index dietary intervention during pregnancy

The aim of this analysis was to examine the effect of a low GI dietary intervention during pregnancy on child outcomes^(12)^. The quality and quantity of carbohydrates influence glycaemic responses, and the GI scale was designed as a method for quantifying the ability of different carbohydrate foods to raise blood glucose concentrations^(16)^. High GI foods, such as potatoes and refined grains, are broken down rapidly in the body causing sharp peaks in blood glucose and insulin responses following food ingestion^(17)^. In contrast, low GI foods, such as legumes and whole grains, have a slower and smaller effect on postprandial blood glucose levels and insulin response because they are absorbed more slowly in the body and release glucose gradually into the bloodstream^(17)^. Therefore, the rationale of following a low GI diet in pregnancy for the purpose of the ROLO study was to regulate the amount of blood glucose supply to the developing fetus.

Women who were randomised to the intervention group attended a 2-h group education session with a research dietitian 2 weeks after randomisation. Attendees had a mean (sd) gestation of 15·7 (3·0) weeks. The research dietitian initially advised on general healthy eating in pregnancy in line with the food pyramid. Guidance was provided on the recommended daily portions of carbohydrates, fruit and vegetables, dairy foods, meat and fish and limited intake of foods high in fat or sugar. The advice lead onto the principles of a low GI diet; what it means, the concept and the rationale for adherence in pregnancy. The advice focused on carbohydrate foods (sugars and starches) and other sources of sugar (fructose in fruit, lactose in milk and yogurts). Participants were encouraged to choose as many low GI foods as possible and to exchange high GI carbohydrate foods for low GI alternatives. Participants were given information leaflets about the GI which included a list of low and high GI foods, low GI recipes, snack options, factsheets and tips (see online Supplementary Material).

The recommended diet was eucaloric and was designed to meet the guidelines for pregnant women^(18)^. The dietary education session did not cover information on glycaemic load (GL) to prevent confusion. GL is a separate measure to GI, which provides a more accurate estimation of a food’s real-life impact on postprandial glycaemia throughout the day^(19)^. GL accounts for the quality of the carbohydrate-containing food (i.e. GI) and the quantity consumed of that food (weight), to estimate how increased and prolonged glycaemia will be when ingesting a specific amount of carbohydrate-rich food^(19)^. Most high GI foods will also have a high GL in a standard serving size; however, moderate GI foods can generate a high GL based on the density or if it is consumed in excess^(19)^. Examples of high GL dietary patterns have been described previously^(17)^. The research dietitian met with intervention subjects again at 28 and 34 weeks’ gestation for brief reinforcement of the low GI diet. Compliance and acceptability of the intervention were assessed at 34 weeks’ gestation using five-point Likert scales. Women in the control group received routine antenatal care, which did not include any formal dietary advice.

#### Maternal sugar intake, carbohydrate intake and carbohydrate quality during pregnancy

This analysis also aimed to examine associations of sugar and carbohydrate intakes, along with the GI and GL of the woman’s diet, during pregnancy with child outcomes (regardless of intervention). Total sugar intake was of particular interest as a proxy for excess-free-fructose intake, previously postulated as a potential cause of asthma and atopy^(9,10)^. It is fructose that occurs when the fructose-to-glucose ratio exceeds 1:1, as found in apples, apple juice, watermelons, mangoes and high fructose corn syrup, that is associated with fructose malabsorption and gut dysbiosis. Data on maternal diet were collected once in each trimester using 3-d food diaries from women in both trial arms. All participants were requested to record any food and beverages consumed over three consecutive days during each trimester of pregnancy, which included two weekdays and one weekend day. In brief, dietary data were entered into the dietary analysis software NetWISP (version 3·0, Tinuviel Software) by a research dietitian using household measures and average portion sizes from the UK Food Standards Agency^(20)^. The NetWISP food composition database was derived from the 6^th^ edition of McCance and Widdowson’s Food Composition Tables^(21)^. Mean daily intake of macronutrients and micronutrients was generated for each time point in pregnancy, as well as the GI and GL of the woman’s diet. A total of 1397 food and beverage items were used to calculate sugar and carbohydrate intakes (see online Supplementary Material). Items with zero or trace carbohydrates were excluded. GI values were determined using the 2008 International Tables of Glycaemic Index Values along with other published GI values^(22,23)^. The GL was calculated as the mathematical product of the GI of a food and its carbohydrate content in grams divided by 100. Sugar and carbohydrate intakes were adjusted for energy intake at each trimester using the residual method^(24)^. Sugar and carbohydrate intakes, along with GI and GL, in each trimester were averaged to obtain mean values through pregnancy.

### Outcome assessment

**Fig. 1.**
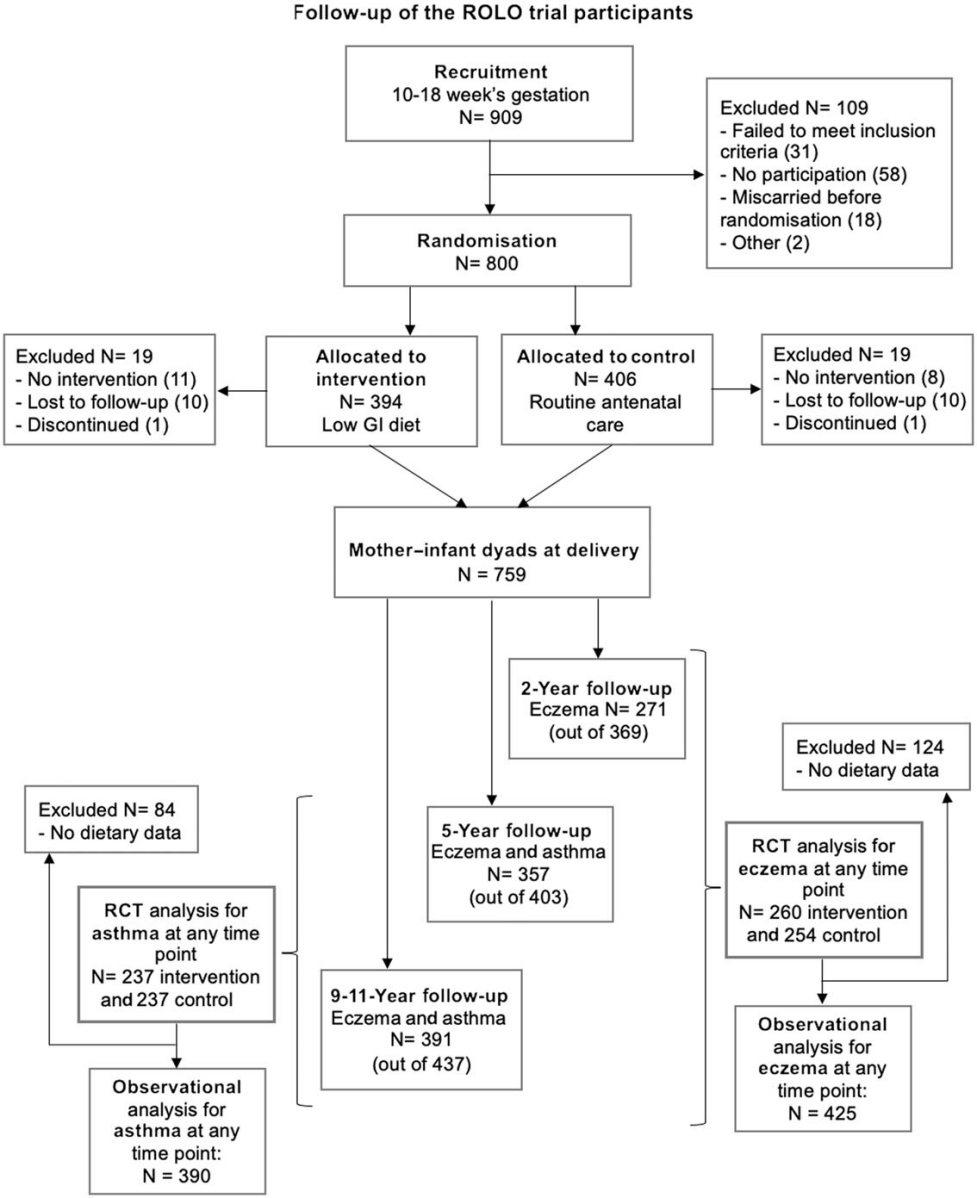
Flow diagram showing loss to follow-up of ROLO trial participants and reasons for exclusion. GI, glycaemic index; RCT, randomised control trial; ROLO, Randomised cOntrol trial of LOw glycaemic index diet in pregnancy *v*. no dietary intervention to prevent recurrence of macrosomia.

Of the total 759 mother–infant dyads included in the primary analysis of the ROLO trial, 369 (48·6 %), 403 (53·0 %) and 437 (57·5 %) participated in 2-year, 5-year and 9–11-year follow-up studies, respectively (see Fig. 1). All mothers who returned for follow-up when the study child was 2, 5 and 9–11 years were asked to complete a questionnaire adapted from the SLAN 2007 lifestyle habits questionnaire (Survey of Lifestyle, Attitudes and Nutrition in Ireland), which included questions on child health^(25)^. The question was phrased as ‘does your child have any ongoing problems, tick all that apply’; the list included whether their child had experienced asthma or eczema. At the 5- and 9–11-year follow-ups children were defined as having current doctor-diagnosed asthma if mothers said their child had ongoing problems with asthma diagnosed by a doctor. At the 2, 5 and 9–11-year follow-ups children were defined as having current doctor-diagnosed eczema if mothers said their child had ongoing problems with eczema diagnosed by a doctor. Reported asthma at 2 years of age was not included because an accurate diagnosis of asthma is difficult in children up to 5 years of age^(26)^. Responses were collated to analyse outcomes at ‘any time point’ in childhood to increase statistical power and separately at each follow-up.

### Statistical analysis

All statistical analyses were performed using Statistical Software for Social Sciences (SPSS) version 27·0 for Mac (Macintosh). The normality of data was assessed using Kolmogorov–Smirnov tests and visually. We used independent *t*-tests, Mann–Whitney *U* tests or *χ* ^2^ tests as appropriate for univariate analysis. Logistic regression models were used to assess the effect of the trial intervention on child asthma and eczema, using a per protocol analysis approach. Of the 800 women who were randomised, those who were non-compliant, lost to follow-up or discontinued with the study prior to delivery and did not have available data on child outcomes were excluded. Therefore, in this secondary analysis, a per protocol approach was chosen to investigate an etiological question among those who completed the trial until delivery and had available data on outcomes in childhood. The intention-to-treat principle was inappropriate due to the loss of participants without outcome assessments. For analyses of the randomised intervention, we chose to compare specific baseline characteristics of interest between trial arms (intervention *v*. control) in participants with follow-up data, based on their known associations with the outcomes^(27)^. Characteristics were analysed separately for each outcome and time point due to differences in sample size to mitigate the differences between the two arms. Follow-up analyses of randomised trials reduce the likelihood of unmeasured confounding; however, confounding variables that significantly differed were included in logistic regression models along with sex of child (male, yes/no) and age of child at follow-up (years) (see Table 1; online Supplementary Tables 1–3). The interaction effect of maternal education on the associations between trial arm and child outcomes was also included in the logistic regression models. We stratified by maternal education (dichotomised into those having completed education from a higher education institute and those who did not)^(28)^. The rationale for this sub-analysis was based on previous findings that mothers who completed tertiary education responded better to the low GI dietary intervention compared with mothers with lower educational attainment, regardless of neighbourhood affluence^(28)^. Sensitivity analyses were performed in the randomised trial analyses to test the effect of adjusting for potential mediators that differed between the two arms (birthweight and gestational age). In observational analyses, multivariate logistic regression models were used to analyse the relations of sugar and carbohydrate intake, along with GI and GL, in pregnancy to child outcomes. The selection of potential covariates to include in observational multivariable logistic regression models was informed by the literature^(27)^ and by a directed acyclic graph, separately for asthma (see online Supplementary Fig. 1) and eczema outcomes (see online Supplementary Fig. 2). Covariates included in the models were mean total energy intake during pregnancy (kcal/day), original trial group (intervention, yes/no), maternal ethnicity (White Irish, yes/no), maternal education level (three categories), smoking in pregnancy (yes/no), age at delivery (years), sex of child (male, yes/no), age of child at follow-up (years), gestational weight gain (GWG) (three categories)^(29)^, gestational age at delivery (days) and birthweight (kg). We analysed each dietary component in quartiles as a categorical variable using the lowest quartile as reference. We tested for linear trends across quartiles by assigning median values to each of the four categories and then including it as a continuous variable in the models (i.e. per quartile effect).

**Table 1.** Maternal child characteristics from the ROLO trial for those with asthma outcomes at any time point

	Total (*N* 474)		Intervention (*N* 237)		Control (*N* 237)		*P* value
	Mean/Median/n	sd/(IQR)/%	Mean/Median/n	sd/(IQR)/%	Mean/Median/n	sd/(IQR)/%	
*Maternal characteristics*							
Age at delivery (years)	33·3	(30·5, 35·6)	33·2	(23·1, 28·4)	33·4	(30·9, 35·6)	0·50
HP index	7·20	(0·60, 12·1)	7·00	(0·45, 10·0)	7·30	(0·70, 12·6)	0·80
Early pregnancy BMI (kg/m^2^)	25·5	(23·3, 28·0)	25·4	(23·1, 28·4)	25·7	(23·3, 28·0)	0·76
*Gestational weight gain*							
Inadequate, *n* (%)	65	13·7	37	15·6	28	11·8	0·43
Adequate, *n* (%)	142	30·0	75	31·6	67	28·3	
Excessive, *n* (%)	186	39·2	87	36·7	99	41·8	
Missing, *n* (%)	81	17·1	38	16·0	43	18·1	
*Smoking in pregnancy*							
Yes, *n* (%)	14	3·00	10	4·20	4	1·70	0·10
No, *n* (%)	460	97·0	227	95·8	233	98·3	
*Education level*							
Completed tertiary level, *n* (%)	245	51·7	122	51·5	123	51·9	0·72
Completed some tertiary level, *n* (%)	89	18·8	41	17·3	48	20·3	
Any secondary education, *n* (%)	80	16·9	44	18·6	36	15·2	
Missing, *n* (%)	60	12·7	30	12·7	30	12·7	
*Ethnicity*							
White Irish, *n* (%)	438	92·4	222	93·7	216	91·1	0·30
Other, *n* (%)	36	7·60	15	6·30	21	8·90	
*Child characteristics*							
*Child sex*							
Male, *n* (%)	236	49·8	120	50·6	116	48·9	0·71
Female, *n* (%)	238	50·2	117	49·4	121	51·1	
Birthweight (kg)	4·02	0·46	4·06	0·47	3·98	0·45	0·04
Gestational age at delivery (days)	283	(277, 288)	284	(278, 289)	282	(277, 288)	0·08
Age at 5-year follow-up (years)	5·17	(5·08, 5·21)	5·17	(5·08, 5·21)	5·17	(5·09, 5·21)	0·40
Age at 9–11-year follow-up (years)	9·88	(9·30, 10·2)	9·88	(9·27, 10·2)	9·88	(9·32, 10·2)	0·44

ROLO, Randomised cOntrol trial of LOw glycaemic index diet in pregnancy *v*. no dietary intervention to prevent recurrence of macrosomia; HP, Hasse and Pratschke index. Results are presented as mean (sd standard deviation) for normally distributed variables, median (IQR interquartile range 25th, 75th percentile) for non-normally distributed variables and *n* (%) for categorical variables.

## Results

### Maternal and child characteristics in the Randomised cOntrol trial of a LOw glycaemic index diet in pregnancy to prevent macrosomia trial

Cohort characteristics for those with asthma outcomes at any time point (*n* 474) according to intervention arm are shown in Table 1. The majority of mothers were White Irish (*n* 438) and 51·7 % had completed tertiary level education. Of those included in this analysis, 50 % of mothers were in the intervention group and 49·8 % of the children were male. Among children with available information, 8 % had doctor-diagnosed asthma at 5 or 9–11 years, 7 % at 5 years and 7·7 % at 9–11 years.

### Analyses of the randomised intervention and child outcomes

We did not find strong evidence that a low GI dietary intervention during pregnancy reduced the risk of asthma or eczema in childhood. However, a lower proportion of children whose mothers had received the intervention developed asthma overall and at each time point compared with usual care. At age 5, there was a suggestion of a reduction in asthma at 5 years of age in children whose mothers had received the intervention compared with usual care in the adjusted model (adjusted OR 0·46 (95 % CI 0·19, 1·09); *P* = 0·08) (Table 2). Adjustment for potential mediators (birthweight and gestational age) in sensitivity analyses yielded similar conclusions in all models (online Supplementary Table 4).

**Table 2.** Associations between ROLO trial intervention arms in pregnancy and child outcomes

Trial group	Asthma				Eczema			
	Control	Intervention			Control	Intervention		
	Ref	OR	95 % CI	*P*-value	Ref	OR	95 % CI	*P*-value
Any time point								
Cases/non-cases	21/216	17/220			30/224	36/224		
Unadjusted	1·00	0·79	0·40, 1·54	0·50	1·00	1·20	0·71, 2·01	0·49
Adjusted*,†	1·00	0·79	0·40, 1·54	0·49	1·00	1·18	0·70, 2·00	0·52
2 years								
Cases/non-cases	–	–		–	16/113	18/124		
Unadjusted	–	–		–	1·00	1·02	0·49, 2·10	0·95
Adjusted‡	–	–		–	1·00	1·00	0·48, 2·08	0·99
5 years								
Cases/non-cases	9/177	16/155			14/157	19/167		
Unadjusted	1·00	0·49	0·21, 1·14	0·10	1·00	1·27	0·61, 2·63	0·51
Adjusted§	1·00	0·46	0·19, 1·09	0·08	1·00	1·25	0·60, 2·61	0·53
9–11 years								
Cases/non-cases	17/178	13/183			9/186	9/187		
Unadjusted	1·00	0·74	0·35, 1·57	0·44	1·00	0·99	0·38, 2·56	0·99
Adjusted^||^	1·00	0·71	0·33, 1·52	0·39	1·00	0·98	0·38, 2·55	0·98

ROLO, Randomised cOntrol trial of LOw glycaemic index diet in pregnancy *v*. no dietary intervention to prevent recurrence of macrosomia; Ref, Reference group.

Values determined using logistic regression.

* Asthma model: adjusted for child sex, age at follow-up.

† Eczema model: adjusted for maternal smoking in pregnancy, child sex, age at follow-up.

‡ Model adjusted for child sex, age at follow-up.

§ Models adjusted for maternal age at delivery, child sex, age at follow-up.

|| Models adjusted for child sex, age at follow-up.

In addition, there was evidence for effect modification by maternal education at 5 years of age (*P*_interaction_ = 0·045). When we stratified by maternal education, the intervention was associated with a reduction in risk of asthma at 5 years of age in children born to mothers with lower educational attainment but not in those with higher educational attainment (adjusted OR 0·14 (95 % CI 0·02, 0·69); *P* = 0·010 and adjusted OR 1·03 (95 % CI 0·34, 3·13); *P* = 0·94, respectively) (Table 3).

**Table 3.** Associations between ROLO trial intervention arms in pregnancy and child outcomes, stratified by maternal education level

Trial group	Asthma				Eczema			
	Control	Intervention			Control	Intervention		
	Ref	OR	95 % CI	*P*-value	Ref	OR	95 % CI	*P*-value
Incomplete tertiary level education								
Any time point								
Cases/non-cases	12/72	6/79			11/81	12/86		
Unadjusted	1·00	0·45	0·16, 1·27	0·14	1·00	1·02	0·42, 2·45	0·95
Adjusted*,†	1·00	0·44	0·15, 1·26	0·13	1·00	1·01	0·41, 2·48	0·98
2 years								
Cases/non-cases	–	–		–	5/37	4/52		
Unadjusted	–	–		–	1·00	0·56	0·14, 2·26	0·42
Adjusted‡	–	–		–	1·00	0·35	0·07, 1·79	0·21
5 years								
Cases/non-cases	9/47	2/69			6/50	7/64		
Unadjusted	1·00	0·15	0·03, 0·73	0·02	1·00	0·91	0·28, 2·88	0·88
Adjusted§	1·00	0·14	0·02, 0·69	0·01	1·00	0·85	0·26, 2·73	0·78
9–11 years								
Cases/non-cases	10/57	5/61			5/62	5/61		
Unadjusted	1·00	0·46	0·15, 1·45	0·19	1·00	1·01	0·28. 3·68	0·98
Adjusted^||^	1·00	0·47	0·15, 1·49	0·20	1·00	0·98	0·27, 3·59	0·98
Complete tertiary level education								
Any time point								
Cases/non-cases	8/115	11/111			14/117	21/109		
Unadjusted	1·00	1·42	0·55, 3·67	0·46	1·00	1·61	0·78, 3·32	0·20
Adjusted¶,**	1·00	1·42	0·54, 3·71	0·46	1·00	1·70	0·79, 3·70	0·17
2 years								
Cases/non-cases	–	–		–	9/66	13/62		
Unadjusted	–	–		–	1·00	1·53	0·61, 3·85	0·36
Adjusted*†	–	–		–	1·00	1·42	0·53, 3·79	0·48
5 years								
Cases/non-cases	7/93	7/90			5/95	9/88		
Unadjusted	1·00	1·03	0·34, 3·06	0·95	1·00	1·94	0·62, 6·02	0·25
Adjusted*‡	1·00	1·03	0·34, 3·13	0·94	1·00	1·83	0·58, 5·75	0·30
9–11 years								
Cases/non-cases	6/98	8/95			2/102	4/99		
Unadjusted	1·00	1·37	0·46, 4·11	0·57	1·00	2·06	0·36, 11·50	0·41
Adjusted*§	1·00	1·22	0·38, 3·96	0·73	1·00	2·12	0·36, 12·44	0·40

ROLO, Randomised cOntrol trial of LOw glycaemic index diet in pregnancy *v*. no dietary intervention to prevent recurrence of macrosomia; Ref, Reference group.

Values determined using logistic regression.

* Asthma model: adjusted for child sex, age at follow-up.

† Eczema model: adjusted for maternal smoking in pregnancy, HP index, child sex, age at follow-up.

‡ Model adjusted for HP index, child sex, age at follow-up.

§ Models adjusted for maternal age at delivery, child sex, age at follow-up.

|| Models adjusted for child sex, age at follow-up.

¶ Asthma model: adjusted for child sex, age at follow-up.

** Eczema model: adjusted for gestational weight gain, child sex, age at follow-up.

*† Model adjusted for gestational weight gain, child sex, age at follow-up.

*‡ Models adjusted for maternal age at delivery, child sex, age at follow-up.

*§ Models adjusted for gestational weight gain, child sex, age at follow-up.

### Observational analyses of intakes in pregnancy and child outcomes

In the fully adjusted model, there was a positive association between intake of sugar during pregnancy and asthma at any time point (OR per quartile 1·40 (95 % CI 0·99, 1·97); *P*_trend_=0·048) (Table 4). When we analysed each time point, intake of sugar during pregnancy was positively associated only with asthma at 5 years of age (OR per quartile 1·55 (95 % CI 1·00, 2·40), *P*_trend_=0·046) (Table 5).

**Table 4.** Associations between maternal mean sugar intake during pregnancy and child outcomes at any time point

	Quartiles of mean sugar intake during pregnancy (g)							*P* for trend		
	Q1 (28·5g – 76·2g)	Q2 (76·2g – 89·2g)		Q3 (89·3g – 104g)		Q4 (104g – 228g)			Per quartile	
		OR	95 % CI	OR	95 % CI	OR	95 % CI		OR	95 % CI
Asthma at any time point										
Cases/non-cases	6/92	6/94		7/88		13/84				
OR (95 % CI)*	1·00	1·02	0·31, 3·33	1·26	0·40, 3·94	2·33	0·84, 6·41	0·38	1·35	0·96, 1·88
OR (95 % CI)†	1·00	1·18	0·35, 3·95	1·28	0·40, 4·08	2·53	0·89, 7·16	0·07	1·36	0·97, 1·91
OR (95 % CI)‡	1·00	1·13	0·33, 3·77	1·29	0·40, 4·15	2·45	0·86, 6·94	0·07	1·35	0·96, 1·90
OR (95 % CI)§	1·00	1·30	0·38, 4·47	1·51	0·46, 4·95	2·77	0·96, 8·03	0·048	1·40	0·99, 1·97
Eczema at any time point										
Cases/non-cases	14/90	11/101		13/92		17/87				
OR (95 % CI)*	1·00	0·72	0·31, 1·69	0·93	0·41, 2·11	1·24	0·57, 2·67	0·45	1·09	0·85, 1·41
OR (95 % CI)†	1·00	0·81	0·34, 1·92	1·06	0·46, 2·46	1·31	0·59, 2·89	0·40	1·11	0·86, 1·44
OR (95 % CI)‡	1·00	0·79	0·33, 1·90	1·05	0·45, 2·43	1·33	0·59, 2·95	0·38	1·12	0·86, 1·45
OR (95 % CI)§	1·00	0·78	0·32, 1·89	1·12	0·48, 2·64	1·30	0·58, 2·91	0·38	1·12	0·86, 1·45

Q, quartile.

Values determined using multivariate logistic regression.

* Crude model adjusted for mean total energy intake.

† Model 1 adjusted for child sex, age at follow-up, trial group, maternal education, maternal ethnicity, mean total energy intake.

‡ Model 2 adjusted for model 1 + maternal age at delivery, maternal smoking in pregnancy.

§ Model 3 adjusted for model 2 + gestational weight gain, birthweight, gestational age at delivery.

**Table 5.** Associations between maternal mean sugar intake during pregnancy and child outcomes at specific time points

	Quartiles of mean sugar intake during pregnancy (g)									
	Q1	Q2		Q3		Q4			Per quartile	
		OR	95 % CI	OR	95 % CI	OR	95 % CI	*P*_for trend_	OR	95 % CI
2-years										
Eczema										
Cases/non-cases	5/44	9/52		7/63		8/56				
OR (95 % CI)*	1·00	1·57	0·48, 5·07	0·99	0·29, 3·35	1·24	0·38, 4·07	0·61	1·00	0·72, 1·43
OR (95 % CI)†	1·00	2·08	0·62, 7·00	1·21	0·35, 4·19	1·41	0·40, 4·86	0·88	1·02	0·71, 1·48
OR (95 % CI)‡	1·00	2·18	0·64, 7·37	1·24	0·35, 4·32	1·40	0·40, 4·85	0·54	1·02	0·71, 1·47
OR (95 % CI)§	1·00	2·29	0·65, 8·10	1·42	0·38, 5·20	1·40	0·38, 5·10	0·88	1·03	0·70, 1·50
5-years										
Asthma										
Cases/non-cases	3/75	4/71		6/72		9/69				
OR (95 % CI)*	1·00	1·56	0·33, 7·37	2·27	0·54, 9·56	3·18	0·82, 12·30	0·07	1·46	0·97, 2·19
OR (95 % CI)†	1·00	1·61	0·33, 7·69	2·03	0·47, 8·70	3·36	0·84, 13·33	0·07	1·47	0·96, 2·25
OR (95 % CI)‡	1·00	1·55	0·32. 7·43	2·05	0·47, 8·92	3·31	0·82, 13·25	0·07	1·47	0·96, 2·26
OR (95 % CI)§	1·00	1·69	0·34, 8·44	2·17	0·48, 9·77	3·91	0·94, 16·22	0·046	1·55	1·00, 2·40
Eczema										
Cases/no*n*-cases	7/71	3/73		5/73		10/68				
OR (95 % CI)*	1·00	0·45	0·11, 1·84	0·73	0·22, 2·43	1·45	0·52, 4·05	0·32	1·18	0·82, 1·71
OR (95 % CI)†	1·00	0·44	0·10,1·88	0·88	0·25, 3·05	1·48	0·51, 4·30	0·31	1·20	0·82, 1·74
OR (95 % CI)‡	1·00	0·42	0·10, 1·83	0·87	0·25, 3·03	1·51	0·51, 4·45	0·30	1·21	0·83, 1·76
OR (95 % CI)§	1·00	0·40	0·09, 1·76	0·90	0·25, 3·21	1·60	0·52, 4·91	0·25	1·24	0·84, 1·84
9–11 years										
Asthma										
Cases/no*n*-cases	5/71	5/79		5/75		9/73				
OR (95 % CI)*	1·00	0·92	0·25, 3·34	0·96	0·26, 3·48	1·71	0·54, 5·39	0·53	1·21	0·82, 1·77
OR (95 % CI)†	1·00	1·09	0·29, 4·10	1·01	0·27, 3·79	1·84	0·56, 6·01	0·31	1·21	0·82, 1·78
OR (95 % CI)‡	1·00	1·02	0·27, 3·89	1·00	0·26, 3·76	1·77	0·54, 5·85	0·33	1·20	0·81, 1·78
OR (95 % CI)§	1·00	1·31	0·33, 5·19	1·33	0·33, 5·31	2·17	0·63, 7·38	0·21	1·27	0·86, 1·89
Eczema										
Cases/no*n*-cases	4/72	1/83		5/75		5/77				
OR (95 % CI)*	1·00	0·20	0·02, 1·86	1·16	0·30, 4·52	1·22	0·31, 4·78	0·42	1·22	0·75, 1·98
OR (95 % CI)†	1·00	0·14	0·01, 1·44	1·17	0·28, 4·81	1·00	0·24, 4·13	0·49	1·19	0·72, 1·95
OR (95 % CI)‡	1·00	0·13	0·01, 1·30	1·17	0·28, 4·89	0·90	0·21, 3·81	0·54	1·17	0·71, 1·93
OR (95 % CI)§	1·00	0·10	0·00, 1·21	0·82	0·16, 4·07	0·92	0·20, 4·19	0·56	1·16	0·68, 1·98

Q, quartile.

Values determined using multivariate logistic regression.

* Crude model adjusted for mean total energy intake.

† Model 1 adjusted for child sex, age at follow-up, trial group, maternal education, maternal ethnicity, mean total energy intake.

‡ Model 2 adjusted for model 1 + maternal age at delivery, maternal smoking in pregnancy.

§ Model 3 adjusted for model 2 + gestational weight gain, birthweight, gestational age at delivery.

In the fully adjusted model, intake of carbohydrate during pregnancy was positively associated with asthma at any time point in childhood (fully adjusted OR per quartile 1·59 (95 % CI 1·10, 2·30), *P*_trend_ = 0·011) (online Supplementary Table 5). When we analysed follow-up at each time point, intake of carbohydrate during pregnancy was positively associated only with asthma at 9–11 years of age (fully adjusted OR per quartile 1·61 (95 % CI 1·04, 2·49), *P*_trend_ = 0·024) (online Supplementary Table 6).

In the fully adjusted model, mean GI during pregnancy was positively associated with eczema at 9–11 years of age (OR per quartile 1·92 (95 % CI 0·99, 3·74), *P*_trend_ = 0·027); however, mean GL during pregnancy was not associated with eczema at 9–11 years of age (OR per quartile 1·00 (95 % CI 0·57, 1·75), *P*_trend_ = 0·97). While mean GL during pregnancy was positively associated with asthma at any time point (OR per quartile 1·46 (95 % CI 1·00, 2·13), *P*_trend_ = 0·043), mean GI during pregnancy was not associated with asthma at any time point (OR per quartile 0·92 (95 % CI 0·63, 1·32), *P*_trend_ = 0·67) (data not shown).

## Discussion

### Main findings

This study found a suggestion for a reduction in asthma at 5 years of age in children whose mothers had received a low GI dietary intervention during pregnancy compared with usual care. However, amongst mothers with lower educational attainment, the intervention was associated with a reduction in asthma risk in childhood. Also, in keeping with previous birth cohort findings^(9,10)^, maternal intake of sugar during pregnancy was positively associated with the risk of childhood asthma in observational analyses.

### Interpretation

The ROLO low GI intervention resulted in significant reductions in GI, GL and carbohydrate intake in pregnancy^(12,13,30)^, and follow-up of the offspring provided a novel opportunity to test whether previous observational associations linking higher prenatal sugar exposure to increased risk of asthma might be causal. We found a suggestion for a reduced risk of asthma in 5-year-old children born to mothers in the intervention group compared with children born to mothers in the control arm, which strengthens causal inference. However, when we carried out an *a priori* stratified analysis, we found that the intervention reduced childhood asthma risk amongst mothers with lower educational attainment, a strong proxy for social disadvantage^(31)^. Pregnant women with lower educational attainment are more likely to have children with asthma^(31)^, poor dietary intakes and to experience excess GWG^(32)^. Thus, the ROLO low GI dietary intervention may have had a greater impact on reducing childhood asthma risk in this vulnerable subgroup.

To our knowledge, three birth cohort studies have investigated the relation between maternal sugar intake in pregnancy and asthma and atopic outcomes in childhood^(9,10,33)^. In the ALSPAC and Project Viva cohorts, higher free sugar and fructose intakes during pregnancy were associated with increased risk of asthma in mid-childhood^(9,10)^. In contrast, in the Danish National Birth Cohort, Maslova *et al* found no association between the consumption of sugar-sweetened beverages during pregnancy and childhood asthma^(33)^. Our observational associations for sugar intake in pregnancy and childhood asthma risk are in keeping with the ALSPAC and Project Viva findings. In ALSPAC, higher maternal sugar intake was also associated with an increased risk of childhood atopy, as measured by allergen skin testing^(9)^. In the ROLO study, we did not have objective measurements of atopy. We therefore used eczema as a proxy measure of an atopic tendency, but found no association between maternal sugar intake and childhood eczema, although GI was positively associated with eczema at 9–11 years of age.

### Mechanisms

Excess dietary sugars, particularly fructose in its isolated form, are associated with elevated inflammatory markers, C-reactive protein and uric acid in mice and humans^(34–36)^. As mentioned, excess-free-fructose is a major component of apple juice and high fructose corn syrup that is associated with upregulation of aggravated lung allergic inflammation^(37,38)^. Potential underlying mechanisms include upregulation of T-helper type 2 cells (Th2), mucus hypersecretion, increased inflammatory infiltrate and activated receptors of advanced glycation end products in the lung^(35,39–42)^. The direct inflammatory effects of isolated fructose may be explained by adiposity-independent mechanisms^(43)^. Previous research suggests that fetal exposure to a high fibre diet during pregnancy may modulate Th2 immune response and reduce allergic outcomes postnatally^(44)^. However, if the low GI intervention had reduced childhood asthma risk through a reduction in Th2 responses in our cohort, we would have expected to see a reduction in eczema too, and we did not. It is plausible that our mixed findings may be attributed to the consumption of low GI foods that also contain high excess-free-fructose such as apples and pears. Alternatively, there is strong evidence linking gut dysbiosis with the development of asthma^(45)^. Excess-free-fructose can trigger fructose malabsorption-related mechanisms, leading to gut dysbiosis in the gut/lung axis^(46)^. An overabundance of unfavourable microbiota metabolites may also trigger the receptor for advanced glycation end products, a key mediator in the pulmonary inflammatory response^(47)^. It is plausible that gut dysbiosis can be transferred to the fetus during pregnancy via the maternal–fetal gut microbiota axis^(48)^. Thus, maternal dietary factors could have an important role in the modulation of dysbiotic gut microbiota by increasing the production of favourable short-chain fatty acids and promoting a protective T1 phenotype in the fetal lung^(49,50)^.

### Strengths and limitations

A strength of this study is the randomised nature of the intervention in the ROLO pregnancy trial, which reduces the likelihood of confounding which is more likely to have occurred in previous observational studies investigating this hypothesis. Consistent data collection methods for child outcomes were used at each follow-up. For the observational analyses, comprehensive dietary data at three time points in pregnancy were estimated from 3-d food diaries, which is considered a more accurate method than FFQ, and use of nine food diaries overall provides a more accurate estimate of dietary factors throughout pregnancy. Any misclassification of maternal sugar intake in pregnancy is likely to have been random with respect to childhood outcomes, which would tend to push effect estimates towards the null.

Our sample size was not large, and we are therefore likely to have been underpowered to detect modest associations. Another limitation was the substantial loss to follow-up in all three follow-up studies; however, the attrition was not materially different between the two arms of the trial and strategies have been implemented to minimise the attrition rate^(51)^. Our finding that the intervention reduced childhood asthma risk amongst mothers with lower educational attainment but not in those with complete tertiary education should be interpreted with caution. The wide and overlapping confidence intervals observed may indicate an unstable estimate, likely due to the low sample size. In the observational analyses, we cannot rule out the possibility that our main findings arose through unmeasured or residual confounding, although we controlled for a large number of covariates in the regression models. Given the large number of statistical tests in the secondary observational analyses, we acknowledge that some results may have arisen by chance and should therefore be interpreted with caution. However, as the hypotheses we were testing were *a priori*, we have not adjusted for multiple testing.

### Future research and policy implications

This novel study provides stronger evidence that higher sugar intake during pregnancy is associated with an increased risk of asthma among offspring, and an intervention to reduce sugar intake in pregnancy may have potential as a primary prevention strategy. Maternity dietary guidelines focus on short-term maternal implications such as GWG and gestational diabetes mellitus^(52)^, and there is no specific recommendation for the consumption of free sugars during pregnancy^(53)^. Future *de novo* trials of restricted sugar intake during pregnancy may yield confirmatory evidence to inform new guidelines. Public health policies that educate and support pregnant women, especially those with lower educational attainment, to reduce their sugar intake may be beneficial to prevent asthma in their children.

### Conclusion

To conclude, this study suggests that a low GI dietary intervention in pregnancy may reduce the risk of asthma in childhood, especially amongst those born to mothers with lower educational attainment, and further observational evidence that higher maternal sugar intake is associated with higher childhood asthma risk. This new randomised evidence supports a causal link and thus has important implications for the primary prevention of childhood asthma. Our findings may strengthen evidence-based maternity dietary guidelines related to sugar intake.

## Supporting information

Callanan et al. supplementary material Callanan et al. supplementary material

## Data Availability

The datasets used and/or analysed during the current study are available from the corresponding author on reasonable request.

## Supplementary material

For supplementary material/s referred to in this article, please visit https://doi.org/10.1017/S0007114524001612

## References

[1] García-Marcos L , Asher MI , Pearce N , et al. (2022) The burden of asthma, hay fever and eczema in children in 25 countries: GAN phase I study. Eur Respir J 60, 2102866. 35144987 10.1183/13993003.02866-2021 PMC9474895

[2] Duijts L (2012) Fetal and infant origins of asthma. Eur J Epidemiol 27, 5–14. 22350146 10.1007/s10654-012-9657-y PMC3292726

[3] Venter C , Agostoni C , Arshad SH , et al. (2020) Dietary factors during pregnancy and atopic outcomes in childhood: a systematic review from the european academy of allergy and clinical immunology. Pediatr Allergy Immunol 31, 889–912. 32524677 10.1111/pai.13303 PMC9588404

[4] Beckhaus AA , Garcia-Marcos L , Forno E , et al. (2015) Maternal nutrition during pregnancy and risk of asthma, wheeze, and atopic diseases during childhood: a systematic review and meta-analysis. Allergy 70, 1588–1604. 26296633 10.1111/all.12729

[5] Mensink-Bout SM , van Meel ER , de Jongste JC , et al. (2022) Maternal diet in pregnancy and child’s respiratory outcomes: an individual participant data meta-analysis of 18 000 children. Eur Respir J 59, 2101315. 34503987 10.1183/13993003.01315-2021 PMC9030071

[6] Brustad N , Bønnelykke K & Chawes B (2023) Dietary prevention strategies for childhood asthma. Pediatr Allergy Immunol 34, e13984. 37492917 10.1111/pai.13984

[7] Litonjua AA , Carey VJ , Laranjo N , et al. (2020) Six-year follow-up of a trial of antenatal vitamin D for asthma reduction. N Engl J Med 382, 525–533. 32023372 10.1056/NEJMoa1906137 PMC7444088

[8] Brustad N , Eliasen AU , Stokholm J , et al. (2019) High-dose vitamin d supplementation during pregnancy and asthma in offspring at the age of 6 years. Jama 321, 1003–1005. 30860552 10.1001/jama.2019.0052 PMC6439670

[9] Bédard A , Northstone K , Henderson AJ , et al. (2017) Maternal intake of sugar during pregnancy and childhood respiratory and atopic outcomes. Eur Respir J 50, 1700073. 28679610 10.1183/13993003.00073-2017 PMC5540678

[10] Wright LS , Rifas-Shiman SL , Oken E , et al. (2018) Prenatal and early life fructose, fructose-containing beverages, and midchildhood asthma. Ann Am Thorac Soc 15, 217–224. 29219619 10.1513/AnnalsATS.201707-530OC PMC5802621

[11] Shaheen SO , Gissler M , Devereux G , et al. (2020) Maternal iron supplementation in pregnancy and asthma in the offspring: follow-up of a randomised trial in Finland. Eur Respir J 55, 1902335. 32139461 10.1183/13993003.02335-2019

[12] Walsh JM , McGowan CA , Mahony R , et al. (2012) Low glycaemic index diet in pregnancy to prevent macrosomia (ROLO study): randomised control trial. BMJ 345, e5605. 22936795 10.1136/bmj.e5605 PMC3431285

[13] McGowan CA , Walsh JM , Byrne J , et al. (2013) The influence of a low glycemic index dietary intervention on maternal dietary intake, glycemic index and gestational weight gain during pregnancy: a randomized controlled trial. Nutr J 12, 1–9. 24175958 10.1186/1475-2891-12-140 PMC4176103

[14] Horan MK , Donnelly JM , McGowan CA , et al. (2016) The association between maternal nutrition and lifestyle during pregnancy and 2-year-old offspring adiposity: analysis from the ROLO study. J Public Health 24, 427–436. 10.1007/s10389-016-0740-9 PMC5025498 27695668

[15] Callanan S , Yelverton CA , Geraghty AA , et al. (2021) The association of a low glycaemic index diet in pregnancy with child body composition at 5 years of age: a secondary analysis of the ROLO study. Pediatr Obes 16, e12820. 34080318 10.1111/ijpo.12820

[16] Jenkins DJ , Wolever T , Taylor RH , et al. (1981) Glycemic index of foods: a physiological basis for carbohydrate exchange. Am J Clin Nutr 34, 362–366. 6259925 10.1093/ajcn/34.3.362

[17] Chang KT , Lampe JW , Schwarz Y , et al. (2012) Low glycemic load experimental diet more satiating than high glycemic load diet. Nutr Cancer 64, 666–673. 22564018 10.1080/01635581.2012.676143 PMC3762696

[18] Health Service Executive (2006) Healthy Eating During Pregnancy. Dublin, Ireland: Health Service Executive.

[19] Bell SJ & Sears B (2003) Low-glycemic-load diets: impact on obesity and chronic diseases. Crit Rev Food Sci Nutr 43, 357–377. 12940416 10.1080/10408690390826554

[20] Crawley H , Patel S , Mills A , et al. (2002) Food Portion Sizes. London, UK: Stationary Office.

[21] Royal Society of Chemistry (2002) McCance and Widdowson’s the Composition of Foods, 6 th ed. Cambridge, UK: Royal Society of Chemistry.

[22] Levis SP , McGowan CA & McAuliffe FM (2011) Methodology for adding and amending glycaemic index values to a nutrition analysis package. Br J Nutr 105, 1117–1132. 21144094 10.1017/S0007114510004769

[23] Atkinson FS , Foster-Powell K & Brand-Miller JC (2008) International tables of glycemic index and glycemic load values: 2008. Diabetes Care 31, 2281–2283. 18835944 10.2337/dc08-1239 PMC2584181

[24] Willet W (2013) Implications of total energy intake for epidemiologic analysis. In Nutritional Epidemilogy, 3rd ed. Oxford, USA: Oxford University Press.

[25] Harrington J , Perry I , Lutomski J , et al. (2008) SLÁN 2007: Survey of Lifestyle, Attitudes and Nutrition in Ireland. Dietary Habits of the Irish Population. Dublin: The Stationery Office.

[26] Martin J , Townshend J & Brodlie M (2022) Diagnosis and management of asthma in children. BMJ Paediatr Open 6, e001277. 10.1136/bmjpo-2021-001277 PMC9045042 35648804

[27] Nurmatov U , Nwaru BI , Devereux G , et al. (2012) Confounding and effect modification in studies of diet and childhood asthma and allergies. Allergy 67, 1041–1059. 22712878 10.1111/j.1398-9995.2012.02858.x

[28] O’Brien EC , Alberdi G , Geraghty AA , et al. (2017) Lower education predicts poor response to dietary intervention in pregnancy, regardless of neighbourhood affluence: secondary analysis from the ROLO randomised control trial. Public Health Nutr 20, 2959–2969. 28807059 10.1017/S1368980017001951 PMC10261293

[29] Institute of Medicine (US) and National Research Council (US), Committee to Re-examine IOM Pregnancy Weight Guidelines (2009) Weight Gain During Pregnancy: Reexamining the Guidelines. Washington (DC): National Academies Press (US).

[30] Horan MK , McGowan CA , Gibney ER , et al. (2016) Maternal nutrition and glycaemic index during pregnancy impacts on offspring adiposity at 6 months of age—analysis from the ROLO randomised controlled trial. Nutrients 8, 7. 26742066 10.3390/nu8010007 PMC4728621

[31] Lewis KM , Ruiz M , Goldblatt P , et al. (2017) Mother’s education and offspring asthma risk in 10 European cohort studies. Eur J Epidemiol 32, 797–805. 28929268 10.1007/s10654-017-0309-0 PMC5662657

[32] O’Brien EC , Alberdi G & McAuliffe FM (2018) The influence of socioeconomic status on gestational weight gain: a systematic review. J Public Health 40, 41–55. 10.1093/pubmed/fdx038 28398550

[33] Maslova E , Strøm M , Olsen SF , et al. (2013) Consumption of artificially-sweetened soft drinks in pregnancy and risk of child asthma and allergic rhinitis. PLOS ONE 8, e57261. 23460835 10.1371/journal.pone.0057261 PMC3584110

[34] Aeberli I , Gerber PA , Hochuli M , et al. (2011) Low to moderate sugar-sweetened beverage consumption impairs glucose and lipid metabolism and promotes inflammation in healthy young men: a randomized controlled trial. Am J Clin Nutr 94, 479–485. 21677052 10.3945/ajcn.111.013540

[35] Kierstein S , Krytska K , Kierstein G , et al. (2008) Sugar consumption increases susceptibility to allergic airway inflammation and activates the innate immune system in the lung. J Allergy Clin Immunol 121, S196.

[36] Johnson RJ , Nakagawa T , Sanchez-Lozada LG , et al. (2013) Sugar, uric acid, and the etiology of diabetes and obesity. Diabetes 62, 3307–3315. 24065788 10.2337/db12-1814 PMC3781481

[37] DeChristopher LR & Tucker KL (2020) Excess free fructose, apple juice, high fructose corn syrup and childhood asthma risk – the national children’s study. Nutr J 19, 60. 32576181 10.1186/s12937-020-00578-0 PMC7313206

[38] DeChristopher LR , Uribarri J & Tucker KL (2016) Intakes of apple juice, fruit drinks and soda are associated with prevalent asthma in US children aged 2–9 years. Public Health Nutr 19, 123–130. 25857343 10.1017/S1368980015000865 PMC10271120

[39] Musiol S , Harris CP , Karlina R , et al. (2023) Dietary digestible carbohydrates are associated with higher prevalence of asthma in humans and with aggravated lung allergic inflammation in mice. Allergy 78, 1218–1233. 36424672 10.1111/all.15589

[40] Kool M , Willart MA , van Nimwegen M , et al. (2011) An unexpected role for uric acid as an inducer of T helper 2 cell immunity to inhaled antigens and inflammatory mediator of allergic asthma. Immun 34, 527–540. 10.1016/j.immuni.2011.03.015 21474346

[41] Yonchuk JG , Silverman EK , Bowler RP , et al. (2015) Circulating soluble receptor for advanced glycation end products (sRAGE) as a biomarker of emphysema and the RAGE axis in the lung. Am J Respir Crit Care Med 192, 785–792. 26132989 10.1164/rccm.201501-0137PP

[42] DeChristopher LR , Uribarri J & Tucker KL (2016) Intakes of apple juice, fruit drinks and soda are associated with prevalent asthma in US children aged 2–9 years. Public Health Nutr 19, 123–130. 25857343 10.1017/S1368980015000865 PMC10271120

[43] Singh VP , Aggarwal R , Singh S , et al. (2015) Metabolic syndrome is associated with increased oxo-nitrative stress and asthma-like changes in lungs. PLOS ONE 10, e0129850. 26098111 10.1371/journal.pone.0129850 PMC4476757

[44] Thorburn AN , McKenzie CI , Shen S , et al. (2015) Evidence that asthma is a developmental origin disease influenced by maternal diet and bacterial metabolites. Nat Commun 6, 7320 26102221 10.1038/ncomms8320

[45] Barcik W , Boutin RCT , Sokolowska M , et al. (2020) The role of lung and gut microbiota in the pathology of asthma. Immun 52, 241–255. 10.1016/j.immuni.2020.01.007 PMC7128389 32075727

[46] DeChristopher LR (2024) 40 years of adding more fructose to high fructose corn syrup than is safe, through the lens of malabsorption and altered gut health–gateways to chronic disease. Nutr J 23, 1–19. 38302919 10.1186/s12937-024-00919-3 PMC10835987

[47] Oczypok EA , Perkins TN & Oury TD (2017) All the “RAGE” in lung disease: the receptor for advanced glycation endproducts (RAGE) is a major mediator of pulmonary inflammatory responses. Paediatr Respir Rev 23, 40–49. 28416135 10.1016/j.prrv.2017.03.012 PMC5509466

[48] Sajdel-Sulkowska EM (2023) The impact of maternal gut microbiota during pregnancy on fetal gut-brain axis development and life-long health outcomes. Microorganisms 11, 2199. 37764043 10.3390/microorganisms11092199 PMC10538154

[49] Alsharairi NA (2020) The infant gut microbiota and risk of asthma: the effect of maternal nutrition during pregnancy and lactation. Microorganisms 8, 1119. 32722458 10.3390/microorganisms8081119 PMC7466123

[50] Gray LE , O’Hely M , Ranganathan S , et al. (2017) The maternal diet, gut bacteria, and bacterial metabolites during pregnancy influence offspring asthma. Front Immunol 8, 365. 28408909 10.3389/fimmu.2017.00365 PMC5374203

[51] O’Brien EC , Geraghty AA & McAuliffe FM (2017) Successful strategies to improve follow-up for longitudinal birth cohort studies. Contemp Clin Trials 57, 8–9. 28302569 10.1016/j.cct.2017.03.003

[52] Institute of Obstetricians and Gynaecologists, The Royal College of Physicians of Ireland (2019) Clinical Practice Guideline: Nutrition for Pregnancy. Dublin, Ireland: Institute of Obstetricians and Gynaecologists.

[53] Gupta A , Singh A , Fernando RL , et al. (2022) The association between sugar intake during pregnancy and allergies in offspring: a systematic review and a meta-analysis of cohort studies. Nutr Rev 80, 904–918. 34432049 10.1093/nutrit/nuab052

