# Supplementary material for: Low glycaemic index diet in pregnancy and child asthma: follow-up of the ROLO trial
Source: Br J Nutr. 2024 Oct 28;135(5):585–95. doi: 10.1017/S0007114524001612 (PMC13161524; doi:10.1017/S0007114524001612)
Supplement: Callanan et al. supplementary material [file S0007114524001612sup001.pdf]

Low glycaemic index diet in pregnancy and child asthma: follow-up of the ROLO trial

Sophie Callanan[1], Mohammad Talaei[2], Anna Delahunt[1], Seif O Shaheen[2,3]*, Fionnuala M McAuliffe[1]*

*Joint senior authors.

Affiliations:

1. UCD Perinatal Research Centre, School of Medicine, University College Dublin, National Maternity Hospital, Dublin, Ireland.
2. Wolfson Institute of Population Health, Barts and The London School of Medicine and Dentistry, Queen Mary University of London, London, UK.
3. Allergy and Lung Health Unit, Melbourne School of Population and Global Health, The University of Melbourne, Victoria, Australia.

Corresponding author:

Professor Fionnuala M McAuliffe, UCD Perinatal Research Centre, School of Medicine, University College Dublin, The National Maternity Hospital, Dublin, Ireland, Telephone: +353 1 637 3216, Fax: +353 1 662 7586, E-mail: fionnuala.mcauliffe@ucd.ie

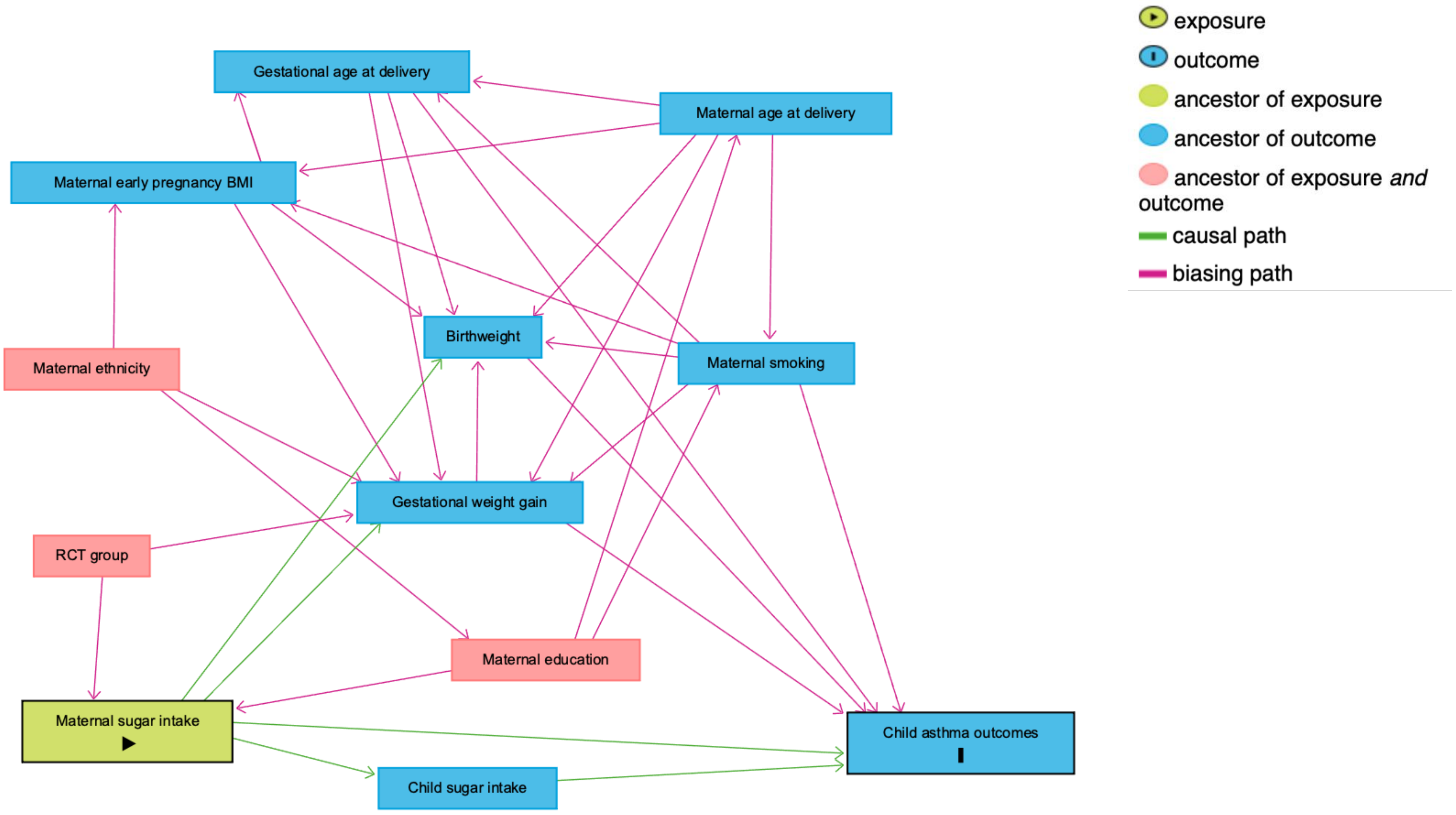

Supplementary Figure 1. Directed acyclic graph of asthma outcomes in childhood created using the online DAGitty tool (1).

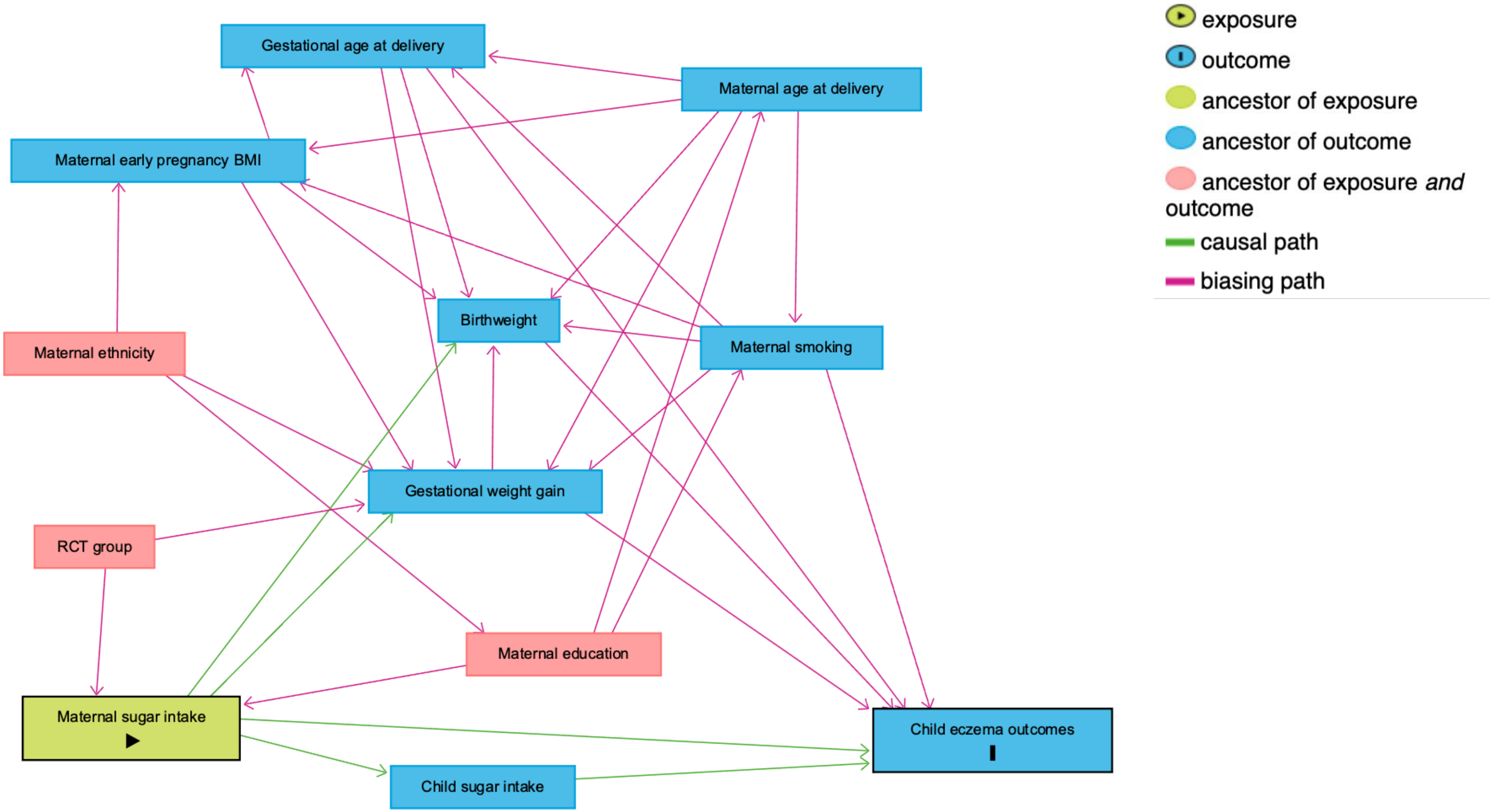

Supplementary Figure 2. Directed acyclic graph of eczema outcomes in childhood created using the online DAGitty tool (1).

Supplementary Table 1. Maternal and child characteristics from the ROLO trial for those with eczema outcomes at any time point.

	Total (N = 514)		Intervention (N = 260)		Control (N = 254)		*P*-value
	Mean / Median / n	SD / (IQR) / %	Mean / Median / n	SD / (IQR) / %	Mean / Median / n	SD / (IQR) / %	
Maternal characteristics							
Age at delivery (years)	33.1	(30.4, 35.5)	32.8	3.95	33.0	3.80	0.49
HP index	7.20	(0.60, 12.4)	6.95	(0.37, 11.9)	7.35	(0.60, 12.7)	0.60
Early pregnancy body mass index (kg/m^2)	25.5	(23.3, 28.0)	25.5	(23.3, 28.5)	25.5	(23.3, 27.9)	0.99
Gestational weight gain							
Inadequate, n (%)	68	13.2	38	14.6	30	11.8	0.48
Adequate, n (%)	150	29.2	81	31.2	69	27.2	
Excessive, n (%)	207	40.3	99	38.1	108	42.5	
Missing, n (%)	89	17.3	42	16.2	47	18.5	
Smoking in pregnancy							
Yes, n (%)	16	3.10	12	4.60	4	1.60	0.047
No, n (%)	498	96.9	248	95.4	250	98.4	
Education level							
Completed tertiary level, n (%)	261	50.8	130	50.0	131	51.6	0.67
Completed some tertiary level, n (%)	101	19.6	48	18.5	53	20.9	
Any secondary education, n (%)	89	17.3	50	19.2	39	15.4	
Missing, n (%)	63	12.3	32	12.3	31	12.2	
Ethnicity							
White Irish, n (%)	471	91.6	242	93.1	229	90.2	0.23
Other, n (%)	43	8.40	18	6.90	25	9.80	
Child characteristics							
Child sex							
Male, n (%)	257	50.0	132	50.8	125	49.2	0.72
Female, n (%)	257	50.0	128	49.2	129	50.8	
Birthweight (kg)	4.02	0.45	4.06	0.46	3.98	0.44	0.05
Gestational age at delivery (days)	283	(277, 288)	284	(277, 289)	282	(277, 288)	0.10
Age at 2-year follow-up (years)	2.06	(2.03, 2.06)	2.06	(2.03, 2.06)	2.06	(2.02, 2.06)	0.96
Age at 5-year follow-up (years)	5.17	(5.09, 5.20)	5.17	(5.09, 5.19)	5.17	(5.09, 5.20)	0.34
Age at 9-11-year follow-up (years)	9.88	(9.36, 10.1)	9.88	(9.33, 10.1)	9.88	(9.36, 10.1)	0.49

Abbreviations: ROLO: Randomised cOntrol trial of LOw glycaemic index diet in pregnancy versus no dietary intervention to prevent recurrence of macrosomia; HP: Hasse and Pratschke index. Results presented as mean (SD standard deviation) for normally distributed variables, median (IQR interquartile range 25th, 75th percentile) for non-normally distributed variables, and n (%) for categorical variables.

Supplementary Table 2. Differences in maternal and child characteristics between trial groups in the ROLO cohort at specific time points.

	Intervention			Control			
	N	Mean / Median / n	SD / (IQR) / %	N	Mean / Median / n	SD / (IQR) / %	*P*-value
**2-year follow-up (N = 271)**							
Maternal characteristics							
Age at delivery (years)	142	33.2	3.84	129	33.2	3.44	0.92
HP index	142	6.62	9.85	129	8.12	9.67	0.21
Early pregnancy body mass index (kg/m^2)	142	25.2	(23.3, 27.9)	129	24.6	(22.6, 27.6)	0.13
Gestational weight gain							
Inadequate, n (%)	142	20	14.1	129	14	10.9	0.10
Adequate, n (%)	142	44	31.0	129	27	20.9	
Excessive, n (%)	142	51	62.0	129	64	49.6	
Missing, n (%)	142	27	16.2	129	24	18.6	
Smoking in pregnancy							
Yes, n (%)	142	4	2.80	129	1	0.80	0.21
No, n (%)	142	138	97.1	129	128	99.2	
Education level							
Completed tertiary level, n (%)	142	75	52.8	129	75	58.1	0.70
Completed some tertiary level, n (%)	142	30	21.1	129	23	17.8	
Any secondary education, n (%)	142	26	18.3	129	19	14.7	
Missing, n (%)	142	11	7.70	129	12	9.30	
Ethnicity							
White Irish, n (%)	142	132	93.0	129	113	87.6	0.14
Other, n (%)	142	10	7.00	129	16	21.4	
Child characteristics							
Birthweight (kg)	142	4.09	0.47	129	4.02	0.42	0.23
Gestational age at delivery (days)	142	284	(277, 289)	129	284	(278, 288)	0.67
**5-year follow-up (N = 357)**							
Maternal characteristics							
Age at delivery (years)	186	32.8	3.99	171	33.8	3.79	0.024
HP index	186	5.80	9.68	171	6.53	9.76	0.53
Early pregnancy body mass index (kg/m^2)	186	25.2	(22.8, 27.6)	171	25.41	(23.3, 27.7)	0.70
Gestational weight gain							
Inadequate, n (%)	186	28	15.1	171	22	12.9	0.75

Adequate, n (%)	186	58	31.2	171	49	28.7	
Excessive, n (%)	186	70	37.6	171	66	38.6	
Missing, n (%)	186	30	16.1	171	34	19.9	
Smoking in pregnancy							
Yes, n (%)	186	7	3.80	171	4	2.30	0.44
No, n (%)	186	179	96.2	171	167	97.7	
Education level							
Completed tertiary level, n (%)	186	97	52.2	171	100	58.5	0.53
Completed some tertiary level, n (%)	186	35	18.8	171	32	18.7	
Any secondary education, n (%)	186	36	19.4	171	24	14.0	
Missing, n (%)	186	18	9.70	171	15	8.80	
Ethnicity							
White Irish, n (%)	186	173	93.0	171	156	91.2	0.66
Other, n (%)	186	13	7.00	171	15	8.80	
Child characteristics							
Birthweight (kg)	186	4.09	0.46	171	3.97	0.42	0.012
Gestational age at delivery (days)	186	284	(278, 290)	171	283	(277, 288)	0.037
**9-11 year follow-up (N = 391)**							
Maternal characteristics							
Age at delivery (years)	196	32.9	4.01	195	32.8	3.76	0.74
HP index	196	6.44	9.12	195	6.03	9.00	0.66
Early pregnancy body mass index (kg/m^2)	196	25.4	(23.1, 28.0)	195	25.7	(23.3, 27.6)	0.81
Gestational weight gain							
Inadequate, n (%)	196	32	16.3	195	19	9.70	0.15
Adequate, n (%)	196	63	32.1	195	56	28.7	
Excessive, n (%)	196	69	35.2	195	83	42.6	
Missing, n (%)	196	32	16.3	195	37	19.0	
Smoking in pregnancy							
Yes, n (%)	196	9	4.60	195	4	2.10	0.16
No, n (%)	196	187	95.4	195	191	97.9	
Education level							
Completed tertiary level, n (%)	196	103	52.6	195	104	53.3	0.91
Completed some tertiary level, n (%)	196	34	17.3	195	38	19.5	
Any secondary education, n (%)	196	32	16.3	195	29	14.9	
Missing, n (%)	196	27	13.8	195	24	12.3	

Ethnicity							
White Irish, n (%)	196	181	92.3	195	179	91.8	0.67
Other, n (%)	196	15	7.7	195	16	8.20	
Child characteristics							
Birthweight (kg)	196	4.06	0.48	195	4.01	0.45	0.30
Gestational age at delivery (days)	196	284	(277, 289)	195	282	(277, 288)	0.12

Abbreviations: ROLO: Randomised cOntrol trial of LOw glycaemic index diet in pregnancy versus no dietary intervention to prevent recurrence of macrosomia; HP: Hasse and Pratschke index. Results presented as mean (SD standard deviation) for normally distributed variables, median (IQR interquartile range 25th, 75th percentile) for non-normally distributed variables, and n (%) for categorical variables.

N = total population with available data; n = frequency.

Supplementary Table 3. Differences in maternal and child characteristics between trial interventions in the ROLO cohort, stratified by maternal education level.

	Intervention			Control			
	N	Mean / Median / n	SD / (IQR) / %	N	Mean / Median / n	SD / (IQR) / %	*P*-value
**Incomplete Tertiary Level Education**							
**Asthma outcomes at any time point (N = 169)**							
Maternal characteristics							
Age at delivery (years)	85	31.8	4.44	84	32.2	3.86	0.57
HP index	85	3.08	10.1	84	5.74	8.79	0.07
Early pregnancy body mass index (kg/m^2)	85	25.8	(23.9, 29.4)	84	25.2	(23.5, 29.6)	0.59
Gestational weight gain							
Inadequate, n (%)	85	10	11.8	84	12	14.3	0.91
Adequate, n (%)	85	24	28.2	84	26	31.0	
Excessive, n (%)	85	37	43.5	84	34	40.5	
Missing, n (%)	85	14	16.5	84	12	14.3	
Smoking in pregnancy							
Yes, n (%)	85	8	9.40	84	3	3.60	0.12
No, n (%)	85	77	90.6	84	81	96.4	
Ethnicity							
White Irish, n (%)	85	82	96.5	84	80	95.2	0.69
Other, n (%)	85	3	3.50	84	4	4.80	
Child characteristics							
Birthweight (kg)	85	4.12	0.48	84	3.92	0.41	0.006
Gestational age at delivery (days)	85	284	(277, 290)	84	281	(274, 288)	0.09
**Eczema outcomes at any time point (N = 190)**							
Maternal characteristics							
Age at delivery (years)	98	31.9	4.30	92	32.3	3.95	0.67
HP index	98	2.72	10.1	92	5.87	9.05	0.025
Early pregnancy body mass index (kg/m^2)	98	25.8	(23.9, 29.3)	92	25.2	(23.3, 29.3)	0.48
Gestational weight gain							
Inadequate, n (%)	98	11	11.2	92	13	14.1	0.87
Adequate, n (%)	98	27	27.6	92	28	30.4	
Excessive, n (%)	98	44	44.9	92	37	40.2	
Missing, n (%)	98	16	16.3	92	14	15.2	

Smoking in pregnancy							
Yes, n (%)	98	10	10.2	92	3	3.30	0.06
No, n (%)	98	88	89.8	92	89	96.7	
Ethnicity							
White Irish, n (%)	98	93	94.9	92	86	93.5	0.68
Other, n (%)	98	5	5.10	92	6	6.50	
Child characteristics							
Birthweight (kg)	98	4.09	0.48	92	3.93	0.40	0.020
Gestational age at delivery (days)	98	282	7.80	92	280	8.48	0.12
**2-year follow-up (N = 98)**							
Maternal characteristics							
Age at delivery (years)	56	32.5	4.19	42	32.8	3.33	0.70
HP index	56	3.35	9.91	42	7.91	9.10	0.022
Early pregnancy body mass index (kg/m^2)	56	26.0	(23.8, 29.8)	42	24.5	(22.7, 28.5)	0.12
Gestational weight gain							
Inadequate, n (%)	56	8	14.3	42	5	11.9	0.97
Adequate, n (%)	56	17	30.4	42	13	31.0	
Excessive, n (%)	56	22	39.3	42	18	42.9	
Missing, n (%)	56	9	16.1	42	6	14.3	
Smoking in pregnancy							
Yes, n (%)	56	4	7.10	42	1	2.40	0.29
No, n (%)	56	52	92.9	42	41	97.6	
Ethnicity							
White Irish, n (%)	56	52	92.9	42	38	90.5	0.67
Other, n (%)	56	4	7.10	42	4	9.50	
Child characteristics							
Birthweight (kg)	56	4.08	0.53	42	3.99	0.37	0.31
Gestational age at delivery (days)	56	281	7.99	42	282	8.54	0.71
**5-year follow-up (N = 127)**							
Maternal characteristics							
Age at delivery (years)	71	31.8	4.57	56	32.6	4.04	0.30
HP index	71	3.90	(-3.90, 10.6)	56	7.10	(1.25, 11.3)	0.23
Early pregnancy body mass index (kg/m^2)	71	25.9	(23.8, 29.3)	56	25.0	(23.5, 30.5)	0.77
Gestational weight gain							
Inadequate, n (%)	71	9	12.7	56	9	16.1	0.26

Adequate, n (%)	71	17	23.9	56	21	37.5	
Excessive, n (%)	71	32	45.1	56	17	30.4	
Missing, n (%)	71	13	18.3	56	9	16.1	
Smoking in pregnancy							
Yes, n (%)	71	6	8.50	56	3	5.40	0.50
No, n (%)	71	65	91.5	56	53	94.6	
Ethnicity							
White Irish, n (%)	71	68	95.8	56	53	94.6	0.77
Other, n (%)	71	3	4.20	56	3	5.40	
Child characteristics							
Birthweight (kg)	71	4.14	0.48	56	3.89	0.38	0.002
Gestational age at delivery (days)	71	285	(278, 290)	56	281	(274, 288)	0.10
**9-11 year follow-up (N = 133)**							
Maternal characteristics							
Age at delivery (years)	66	31.8	4.53	67	31.6	3.95	0.87
HP index	66	3.77	9.64	67	5.44	8.28	0.29
Early pregnancy body mass index (kg/m^2)	66	25.7	(23.9, 29.0)	67	25.1	(23.3, 27.6)	0.54
Gestational weight gain							
Inadequate, n (%)	66	7	10.6	67	8	11.9	0.96
Adequate, n (%)	66	19	28.8	67	19	28.4	
Excessive, n (%)	66	28	42.4	67	30	44.8	
Missing, n (%)	66	12	18.2	67	10	14.9	
Smoking in pregnancy							
Yes, n (%)	66	7	10.6	67	3	4.5	0.18
No, n (%)	66	59	89.4	67	64	95.5	
Ethnicity							
White Irish, n (%)	66	63	95.5	67	65	97.0	0.64
Other, n (%)	66	3	4.50	67	2	3.00	
Child characteristics							
Birthweight (kg)	66	4.10	0.49	67	3.98	0.42	0.14
Gestational age at delivery (days)	66	282	8.27	67	280	8.29	0.08
**Complete Tertiary Level Education**							
**Asthma outcomes at any time point (n = 245)**							
Maternal characteristics							
Age at delivery (years)	122	33.7	3.26	123	33.8	3.57	0.83

HP index	122	8.30	(3.00, 12.4)	123	7.50	(0.80, 12.9)	0.28
Early pregnancy body mass index (kg/m^2)	122	24.7	(22.6, 27.4)	123	25.5	(23.3, 27.6)	0.38
Gestational weight gain							
Inadequate, n (%)	122	20	16.4	123	12	9.8	0.05
Adequate, n (%)	122	46	37.7	123	33	26.8	
Excessive, n (%)	122	37	30.3	123	52	42.3	
Missing, n (%)	122	19	15.6	123	26	21.1	
Smoking in pregnancy							
Yes, n (%)	122	0	0.00	123	0	0.00	-
No, n (%)	122	122	100	123	123	100	
Ethnicity							
White Irish, n (%)	122	110	90.2	123	106	86.2	0.33
Other, n (%)	122	12	9.8	123	17	13.8	
Child characteristics							
Birthweight (kg)	122	4.08	0.43	123	4.02	0.43	0.31
Gestational age at delivery (days)	122	284	(278, 290)	123	283	(279, 288)	0.39
**Eczema outcomes at any time point (N = 261)**							
Maternal characteristics							
Age at delivery (years)	130	33.7	3.30	131	33.6	3.68	0.82
HP index	130	8.30	(3.00, 12.4)	131	7.5	(0.60, 12.9)	0.28
Early pregnancy body mass index (kg/m^2)	130	24.8	(22.6, 27.3)	131	25.5	(23.3, 27.6)	0.82*
Gestational weight gain							
Inadequate, n (%)	130	20	15.4	131	12	9.2	0.025
Adequate, n (%)	130	49	37.7	131	33	25.2	
Excessive, n (%)	130	41	31.5	131	58	44.3	
Missing, n (%)	130	20	15.4	131	28	21.4	
Smoking in pregnancy							
Yes, n (%)	130	0	0.00	131	0	0.00	-
No, n (%)	130	130	100	131	131	100	
Ethnicity							
White Irish, n (%)	130	117	90.0	131	112	85.5	0.27
Other, n (%)	130	13	10.0	131	19	14.5	
Child characteristics							
Birthweight (kg)	130	4.08	0.42	131	4.02	0.43	0.23
Gestational age at delivery (days)	130	284	(277, 290)	131	283	(278, 288)	0.33

**2-year follow-up (N = 150)**							
Maternal characteristics							
Age at delivery (years)	75	33.8	3.40	75	33.4	3.58	0.51
HP index	75	9.11	9.33	75	7.96	9.84	0.47
Early pregnancy body mass index (kg/m^2)	75	24.8	(22.8, 27.1)	75	24.6	(22.2, 27.4)	0.54*
Gestational weight gain							
Inadequate, n (%)	75	10	13.3	75	7	9.30	0.025
Adequate, n (%)	75	25	33.3	75	11	14.7	
Excessive, n (%)	75	25	33.3	75	39	52.0	
Missing, n (%)	75	15	20.0	75	18	24.0	
Smoking in pregnancy							
Yes, n (%)	75	0	0.00	75	0	0.00	-
No, n (%)	75	75	100	75	75	100	
Ethnicity							
White Irish, n (%)	75	69	92.0	75	63	84.0	0.13
Other, n (%)	75	6	8.00	75	12	16.0	
Child characteristics							
Birthweight (kg)	75	4.09	0.43	75	4.04	0.44	0.49
Gestational age at delivery (days)	75	284	(279, 290)	75	284	(279, 288)	0.48
**5-year follow-up (N = 197)**							
Maternal characteristics							
Age at delivery (years)	97	25.2	4.15	100	25.3	3.47	0.36
HP index	97	8.20	(3.05, 12.5)	100	8.5	(1.95, 12.9)	0.76
Early pregnancy body mass index (kg/m^2)	97	24.7	(22.3, 27.1)	100	25.4	(22.6, 27.5)	0.42
Gestational weight gain							
Inadequate, n (%)	97	15	15.5	100	11	11.0	0.07
Adequate, n (%)	97	38	39.2	100	25	25.0	
Excessive, n (%)	97	30	30.9	100	42	42.0	
Missing, n (%)	97	14	14.4	100	22	22.0	
Smoking in pregnancy							
Yes, n (%)	97	0	0.00	100	0	0.00	-
No, n (%)	97	97	100	100	100	100	
Ethnicity							
White Irish, n (%)	97	87	89.7	100	88	88.0	0.71
Other, n (%)	97	10	10.3	100	12	12.0	

Child characteristics							
Birthweight (kg)	97	4.06	0.42	100	4.02	0.43	0.56
Gestational age at delivery (days)	97	284	(279, 290)	100	284	(279, 288)	0.41
**9-11 year follow-up (N = 207)**							
Maternal characteristics							
Age at delivery (years)	103	33.9	3.25	104	33.5	3.54	0.47
HP index	103	8.70	(3.00, 12.5)	104	7.45	(1.95, 12.8)	0.23
Early pregnancy body mass index (kg/m^2)	103	24.8	(22.6, 27.3)	104	25.6	(23.3, 27.6)	0.40
Gestational weight gain							
Inadequate, n (%)	103	19	18.4	104	6.70	6.70	0.008
Adequate, n (%)	103	40	38.8	104	28.8	28.8	
Excessive, n (%)	103	29	28.2	104	42.3	42.3	
Missing, n (%)	103	15	14.6	104	22.1	22.1	
Smoking in pregnancy							
Yes, n (%)	103	0	0.00	104	0	0.00	-
No, n (%)	103	103	100	104	104	100	
Ethnicity							
White Irish, n (%)	103	91	88.3	104	90	86.5	0.69
Other, n (%)	103	12	11.7	104	14	13.5	
Child characteristics							
Birthweight (kg)	103	4.08	0.45	104	4.04	0.43	0.56
Gestational age at delivery (days)	103	284	(277, 289)	104	283	(279, 288)	0.78

Abbreviations: ROLO: Randomised cOntrol trial of LOw glycaemic index diet in pregnancy versus no dietary intervention to prevent recurrence of macrosomia; HP: Hasse and Pratschke index.
Results presented as mean (SD standard deviation) for normally distributed variables, median (IQR interquartile range 25th, 75th percentile) for non-normally distributed variables, and n (%) for categorical variables.
N = total population with available data; n = frequency.
*Log transformed data was used.

Supplementary Table 4. Sensitivity analyses of associations between ROLO trial intervention arms in pregnancy and child outcomes.

Trial Group	Asthma			Eczema		
	Control	Intervention		Control	Intervention	
	Ref	OR (95%CI)	*P*-value	Ref	OR (95%CI)	*P*-value
**Overall**						
**Any time point**						
Cases/non-cases	21/216	17/220		-	-	-
Unadjusted	1.00	0.79 (0.40, 1.54)	0.50	-	-	-
Adjusted[a]	1.00	0.77 (0.39, 1.52)	0.46	-	-	-
**5 years**						
Cases/non-cases	9/177	16/155		14/157	19/167	
Unadjusted	1.00	0.49 (0.21, 1.14)	0.10	1.00	1.27 (0.61, 2.63)	0.51
Adjusted[b]	1.00	0.43 (0.18, 1.03)	0.06	1.00	1.30 (0.61, 2.74)	0.49
**Incomplete Tertiary Level Education**						
**Any time point**						
Cases/non-cases	12/72	6/79		11/81	12/86	
Unadjusted	1.00	0.45 (0.16, 1.27)	0.14	1.00	1.02 (0.42, 2.45)	0.95
Adjusted[c,d]	1.00	0.53 (0.18, 1.54)	0.25	1.00	1.04 (0.42, 2.58)	0.93
**5 years**						
Cases/non-cases	9/47	2/69		6/50	7/64	
Unadjusted	1.00	0.15 (0.03, 0.73)	0.02	1.00	0.91 (0.28, 2.88)	0.88
Adjusted[e]	1.00	0.16 (0.03, 0.85)	0.03	1.00	0.62 (0.17, 2.18)	0.46
**Complete Tertiary Level Education**						
**Any time point**						
Cases/non-cases	8/115	11/111		-	-	-
Unadjusted	1.00	1.42 (0.55, 3.67)	0.46	-	-	-

Adjusted[f]	1.00	1.36 (0.51, 3.59)	0.54	-	-	-
**5 years**						
Cases/non-cases	7/93	7/90		5/95	9/88	
Unadjusted	1.00	1.03 (0.34, 3.06)	0.95	1.00	1.94 (0.62, 6.02)	0.25
Adjusted[g]	1.00	0.97 (0.31, 3.03)	0.97	1.00	2.13 (0.64, 7.04)	0.22

Abbreviations: ROLO: Randomised cOntrol trial of LOw glycaemic index diet in pregnancy versus no dietary intervention to prevent recurrence of macrosomia; Ref: Reference group; OR: Odds ratio; CI: Confidence interval.
Values determined using logistic regression.
[a]Asthma model: adjusted for child sex, age at follow-up, birthweight,
[b]Models adjusted for maternal age at delivery, child sex, age at follow-up, birthweight, gestational age at delivery.
[c]Asthma model: adjusted for child sex, age at follow-up, birthweight.
[d]Eczema model: adjusted for maternal smoking in pregnancy, HP index, child sex, age at follow-up, birthweight.
[e]Models adjusted for maternal age at delivery, child sex, age at follow-up, birthweight, gestational age at delivery.
[f]Asthma model: adjusted for child sex, age at follow-up, birthweight**.**
[g]Models adjusted for maternal age at delivery, child sex, age at follow-up, birthweight, gestational age at delivery.

Supplementary Table 5. Associations between maternal mean carbohydrate intake during pregnancy and child outcomes at any time point.

	**Quartiles of mean carbohydrate intake during pregnancy (g)**					
	Q1 (168g – 217g)	Q2 (217g – 231g)	Q3 (231g – 244g)	Q4 (245g – 341g)	*P* for trend	Per quartile
**Asthma at any time point**						
Cases/non-cases	5/95	7/98	7/98	13/68		
OR (95% CI)[a]	1.00	1.36 (0.41, 4.44)	1.41 (0.43, 4.61)	3.56 (1.21, 10.49)	0.015	1.52 (1.07, 2.16)
OR (95% CI)[b]	1.00	1.39 (0.41, 4.63)	1.37 (0.41, 4.53)	3.62 (1.20, 10.92)	0.017	1.52 (1.06, 2.16)
OR (95% CI)[c]	1.00	1.42 (0.42, 4.76)	1.34 (0.40, 4.50)	3.77 (1.23, 11.52)	0.016	1.53 (1.07, 2.19)
OR (95% CI)[d]	1.00	1.52 (0.44, 5.18)	1.31 (0.38, 4.45)	4.48 (1.42, 14.15)	0.011	1.59 (1.10, 2.30)
**Eczema at any time point**						
Cases/non-cases	13/96	12/98	15/95	15/81		
OR (95% CI)[a]	1.00	0.90 (0.39, 2.08)	1.19 (0.53, 2.66)	1.34 (0.60, 3.00)	0.36	1.12 (0.87, 1.45)
OR (95% CI)[b]	1.00	0.98 (0.41, 2.30)	1.36 (0.60, 3.08)	1.40 (0.61, 3.19)	0.31	1.14 (0.88, 1.48)
OR (95% CI)[c]	1.00	0.97 (0.41, 2.27)	1.34 (0.59, 3.06)	1.39 (0.61, 3.18)	0.32	1.14 (0.88, 1.48)
OR (95% CI)[d]	1.00	0.94 (0.39, 2.21)	1.38 (0.60, 3.17)	1.33 (0.58, 3.07)	0.35	1.13 (0.87, 1.47)

Abbreviations: Q: Quartile; OR: Odds ratio; CI: Confidence interval.
Values determined using multivariate logistic regression.
[a]Crude model adjusted for mean total energy intake.
[b]Model 1 adjusted for child sex, age at follow-up, trial group, maternal education, maternal ethnicity, mean total energy intake.
[c]Model 2 adjusted for Model 1 + maternal age at delivery, maternal smoking in pregnancy.
[d]Model 3 adjusted for Model 2 + gestational weight gain, birthweight, gestational age at delivery.

Supplementary Table 6. Associations between maternal mean carbohydrate intake during pregnancy and child outcomes at specific time points.

	Quartiles of mean carbohydrate intake during pregnancy (g)				*P* for trend	Per quartile
	Q1	Q2	Q3	Q4		
**2-years**						
**Eczema**						
Cases/non-cases	2/46	9/51	10/60	8/58		
OR (95% CI)[a]	1.00	4.03 (0.82, 19.64)	3.85 (0.80, 18.47)	3.09 (0.62, 15.32)	0.35	1.20 (0.83, 1.74)
OR (95% CI)[b]	1.00	4.70 (0.93, 23.60)	4.54 (0.92, 22.44)	3.66 (0.71, 18.85)	0.26	1.26 (0.86, 1.84)
OR (95% CI)[c]	1.00	4.87 (0.86, 24.54)	4.73 (0.95, 23.42)	3.66 (0.71, 18.91)	0.25	1.26 (0.86, 1.85)
OR (95% CI)[d]	1.00	5.13 (1.97, 27.01)	4.93 (0.98, 24.65)	3.80 (0.71, 20.30)	0.23	1.28 (0.86, 1.91)
**5-years**						
**Asthma**						
Cases/non-cases	3/74	6/77	5/81	8/55		
OR (95% CI)[a]	1.00	1.99 (0.47, 8.35)	1.65 (0.37, 7.27)	3.44 (0.86, 13.63)	0.09	1.41 (0.93, 2.13)
OR (95% CI)[b]	1.00	1.98 (0.46, 8.38)	1.50 (0.33, 6.66)	3.23 (0.79, 13.16)	0.13	1.37 (0.90, 2.10)
OR (95% CI)[c]	1.00	1.96 (0.46, 8.31)	1.45 (0.32, 6.56)	3.23 (0.79, 13.23)	0.13	1.37 (0.89, 2.10)
OR (95% CI)[d]	1.00	2.24 (0.50, 9.98)	1.42 (0.30, 6.69)	4.07 (0.94, 17.58)	0.10	1.44 (0.92, 2.24)
**Eczema**						
Cases/non-cases	8/69	4/79	7/79	6/57		
OR (95% CI)[a]	1.00	0.44 (0.12, 1.56)	0.82 (0.28, 2.40)	0.86 (0.28, 2.64)	0.99	0.99 (0.68, 1.45)
OR (95% CI)[b]	1.00	0.50 (0.14. 1.78)	0.91 (0.30, 2.76)	0.97 (0.30, 3.09)	0.85	1.03 (0.70, 1.51)
OR (95% CI)[c]	1.00	0.49 (0.13, 1.76)	0.80 (0.26, 2.50)	0.94 (0.29, 3.02)	0.94	1.01 (0.68, 1.49)
OR (95% CI)[d]	1.00	0.47 (0.13, 1.73)	0.85 (0.26, 2.77)	0.83 (0.25, 2.75)	0.96	0.98 (0.66, 1.45)
**9-11 years**						

**Asthma**						
Cases/non-cases	4/81	5/78	5/79	10/60		
OR (95% CI)[a]	1.00	1.27 (0.33, 4.93)	1.31 (0.33, 4.93)	3.32 (0.99, 11.11)	0.039	1.49 (1.01, 2.22)
OR (95% CI)[b]	1.00	1.26 (0.31, 5.01)	1.24 (0.31, 4.89)	3.18 (0.92, 10.99)	0.05	1.47 (0.98, 2.20)
OR (95% CI)[c]	1.00	1.27 (0.31, 5.06)	1.17 (0.29, 4.68)	3.20 (0.92, 11.15)	0.06	1.46 (0.97, 2.20)
OR (95% CI)[d]	1.00	1.44 (0.35, 5.96)	1.16 (0.28, 4.84)	4.70 (1.26, 17.56)	0.024	1.61 (1.04, 2.49)
**Eczema**						
Cases/non-cases	5/80	2/81	4/80	4/66		
OR (95% CI)[a]	1.00	0.40 (0.07, 2.18)	0.78 (0.20, 3.03)	0.99 (0.25, 3.87)	0.87	1.03 (0.64, 1.65)
OR (95% CI)[b]	1.00	0.38 (0.07, 2.16)	0.78 (0.19, 3.11)	0.98 (0.24, 4.00)	0.89	1.02 (0.63, 1.67)
OR (95% CI)[c]	1.00	0.39 (0.07, 2.17)	0.70 (0.17, 2.92)	0.95 (0.23, 3.91)	0.95	1.00 (0.61, 1.64)
OR (95% CI)[d]	1.00	0.46 (0.07, 2.85)	0.86 (0.18, 3.94)	1.12 (0.23, 5.26)	0.77	1.07 (0.64, 1.79)

Abbreviations: Q: Quartile; OR: Odds ratio; CI: Confidence interval.
Values determined using multivariate logistic regression.
[a]Crude model adjusted for mean total energy intake.
[b]Model 1 adjusted for child sex, age at follow-up, trial group, maternal education, maternal ethnicity, mean total energy intake.
[c]Model 2 adjusted for Model 1 + maternal age at delivery, maternal smoking in pregnancy.
[d]Model 3 adjusted for Model 2 + gestational weight gain, birthweight, gestational age at delivery.

Supplementary Methods

Handling of missing data in analyses

To maximise statistical power, categorical variables with unavailable data were recoded to include a “missing category” for the following; adherence to gestational weight gain guidelines (n = 89); maternal education level (n = 63) for analyses of asthma and eczema outcomes at any time point. A total of 463 imputations were performed for continuous variables with unavailable data. The mean imputation method was used to replace unavailable data with the mean of the available data for the following variables; maternal age at delivery (n = 1); maternal early pregnancy BMI (n = 4); child age at 2-year follow-up (n = 231); child age at 5-year follow-up (n = 125); child age at 9-11-year follow-up (n = 102) for analyses of asthma and eczema outcomes at any time point.

Supplementary References

1. Textor J, van der Zander B, Gilthorpe MS, et al. Robust causal inference using directed acyclic graphs: the R package ‘dagitty’. *Int J Epidemiol.* 2017;45(6):1887-94.

ROLO

Low glycaemic index foods – best choice of carbohydrate

- Apples, oranges, pears, peaches, plums, kiwis, berries
- Beans and lentils
- Most vegetables
- Pasta made from durum wheat
- Basmati rice
- Barley
- Noodles
- Porridge and oat based foods
- Hummus
- Nuts

Medium glycaemic index foods

- Honey
- Couscous
- Baby new potatoes
- Shredded wheat
- Ice cream

High glycaemic index foods – do not eat these foods

- Glucose/sugar, sugary foods & drinks
- White and brown bread
- White and brown rice
- Cornflakes / krispies
- Sugared and malted cereals
- Baked potato
- Mashed potato
- Exotic fruits – banana, mangoes, pineapple, watermelon etc.

A variety of lower glycaemic index versions of bread include pitta bread, rye bread, wraps, granary style bread, pumpernickel bread, and sourdough bread.

An example of a **low glycaemic index meal plan** is outlined below:

Breakfast

- Oat based cereal, e.g. porridge, oatibix
- Low sugar museli, All Bran, Shredded Wheat
- Granary or sourdough bread/toast

Snacks

- Fruit (apples, oranges, pears, peaches, berries, kiwi, plums)
- Natural yogurt
- Popcorn
- Rye bread
- Fruit loaf
- Hummus/cheese/lentil spread on rye or wholegrain crackers
- Oatcakes

Lunch

- Bean or lentil based soup
- Add baked beans to jacket potato or wholegrain toast
- Bean salad
- Add variety with different breads, e.g. pitta bread, wraps, and granary style bread made with mixed grains, rye bread, and pumpernickel bread

Evening meal

- Basmati rice, sweet potato, buckwheat, bulgar wheat, pearl barley, noodles
- Pasta (watch the amount – most people eat too much)
- New potatoes
- Include more beans and pulses (added to soups, stews, and casseroles)

Tips on how to follow a low GI diet

A low GI diet is easy to follow!

Replacing high GI foods with low GI foods in a ‘this for that’ approach will lower the overall GI of each meal, for example eating porridge for breakfast instead of cornflakes, whole-grain bread instead of white bread, or fruit and yogurt in place of cookies.

Remember the GI only applies to foods containing carbohydrate. It is impossible to measure the GI of protein rich foods (meat, eggs, fish, cheese and nuts) or fat rich foods (oils, butter and cream).

A food’s GI does not make it a ‘good’ or ‘bad’ food. Some high GI foods like potatoes are still nutritious foods. Also, many low/medium GI foods like chocolate and crisps that are high in saturated fat and therefore are not particularly healthy. We need to base our food choices on the overall nutritional content of food, taking into account the saturated fat, salt, fibre, energy content, and the GI.

7 Guidelines to low-GI eating

1. *Eat 5 or more servings of fruit and vegetables each day* 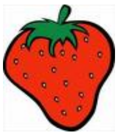 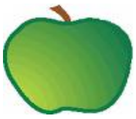

   Aim to have 2 portions of fruit and 3+ portions of vegetables every day. Fruit and vegetables provide us with fibre, water, vitamins and antioxidants which help protect us against colds and flues and numerous diseases (ranging from high blood pressure to cancer). Most fruits have a low GI – particularly apples, oranges, pears, peaches, plums, kiwis and berries. Tropical fruits, such as pineapple, melon, mango and bananas have medium to high GI. Berries have the lowest GI of all fruit so enjoy them by the bowl. Most vegetables have a low GI. Potatoes are the exception; having a high GI. However, new/baby potatoes or sweet potato have lower GI values than old and mashed potatoes.

2. *Eat low GI breads and cereals and grains* 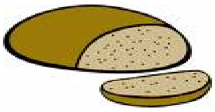 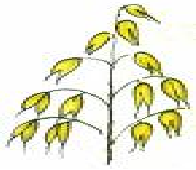

   The type of bread, cereal and grains you eat affect the GI of your diet the most. The best breads to choose include: granary/mixed grain bread, sourdough, rye, pumpernickel, sourdough, pitta, and wraps. Fruit cake has also a low GI due to the fruit content.

   The best cereals to choose are oat based cereals, shredded wheat, no-added sugar muesli or all bran. Grains like pasta, noodles, basmati rice, sweet

potato, new baby potatoes, barley and buckwheat are the lowest GI versions. These foods will keep you fuller and more satisfied for longer.

3. *Eat more legumes, beans, peas, and lentils* 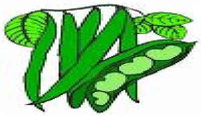 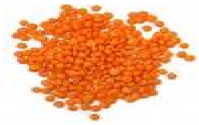

   Legumes (peas, beans, lentils and pulses) have the lowest GI values of almost any other food. They are also high in fibre and are full of nutrients (B vitamins, iron, zinc and magnesium). Legumes are also ‘prebiotics’. This means that they provide food for our gut bacteria and keep our digestive systems healthy.

4. *Eat more nuts and seeds* 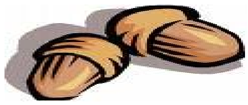

   Even though nuts and seeds are high in fat they mainly contain healthy unsaturated fats, so they are a nutritious substitute for less healthy snacks that are high in saturated (‘bad’) fats such as chocolate and biscuits. Nuts contain lots of vitamin E and selenium; both are powerful antioxidants that protect against premature ageing from UV rays of the sun. Aim to have a small handful of nuts a day which is about 21 almonds/cashews. Flaxseed is useful to sprinkle over cereal or add to baked goods. Nut butters (peanut butter, cashew butter) may be used instead of butter.

5. *Eat more fish and seafood* 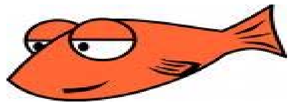

   Fish does not have a GI value as it is a protein food. Fish is an excellent source of protein and healthy fats. Oily fish (salmon, trout, mackerel, sardines and fresh tuna) contain omega 3 fats which are important for your baby’s brain and eye development, but they also protect you from developing heart disease. Some research has linked omega 3 fats with better mood, lower rates of depression and stronger immune function. Aim to have at least one portion of oily fish and one portion of white fish each week. If you dislike fish then an omega 3 supplement is recommended (examples ‘mor-DHA’ or ‘pregnacare’ with omega 3).

6. *Eat lean red meat, poultry and eggs* 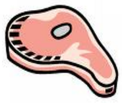 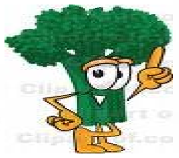 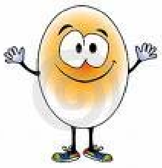

   Meat, chicken and eggs have no GI value as they are a protein food and not carbohydrate. Red meat is the best source of iron (helps in the formation of your baby’s blood). Having healthy levels of iron stores increases your energy levels and improves exercise tolerance. A chronic shortage of iron

leads to anaemia, the symptoms of which include pale skin, excessive tiredness, breathlessness and decreased attention span. Choose at least two portions of red meat each week (beef, lamb). Other sources of iron include green leafy vegetables, egg yolk and darker cuts of poultry.

7. *Eat dairy products* 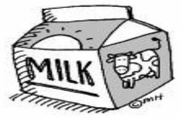

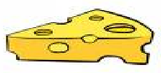
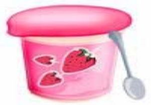

Milk, cheese, yogurt and milk products (milk puddings, custard, ice-cream) are the richest sources of calcium in our diet. Calcium is essential for healthy bones and teeth. It also controls your blood pressure and muscle function. Calcium is particularly important in the last trimester of pregnancy as your baby is laying down their own bones. Try to have 5 servings of dairy products each day (glass of milk, yogurt, and matchbox size of cheese). Remember to count the milk used to make puddings and custards. Ice-cream has a slightly higher GI than milk as there is some sugar added but can be enjoyed in moderation.

ROLO_Foods_for_Carbohydrates_Sugars

food_code	Food _Name
11485	All-Bran
14870	Almonds
14803	Almonds, toasted
2018	Alpen Light cereal bar (fruit)
2006	Alpro Soya Probiotic Dairy free altern to yogurt, w Ca
10256	Ambient Tomato-based sauce for bolognese, FSA
10258	Ambient Tomato-based sauce, lower fat, FSA Sept 2004
10259	Ambient/ fresh Tomato-based sauce + vegetables, FSA
10257	Ambient/Fresh Tomato-based sauce, Napoletana, FSA
10214	American muffins, chocolate, FSA Sept 2004
10215	American muffins, not chocolate, FSA Sept 2004
17531	Apple chutney
14272	Apple juice concentrate, unsweetened
14272	Apple juice concentrate, unsweetened
14271	Apple juice, unsweetened
11278	Apple pie, one crust
11279	Apple pie, pastry top and bottom
11281	Apple pie, wholemeal, pastry top and bottom
17288	Apple sauce, homemade
14006	Apples, cooking, baked with sugar, flesh and skin
14002	Apples, cooking, raw, peeled
14004	Apples, cooking, stewed with sugar
14005	Apples, cooking, stewed without sugar
14012	Apples, eating, average, raw
14014	Apples, eating, average, raw, peeled
14019	Apples, eating, Golden Delicious, raw
14021	Apples, eating, Granny Smith, raw
14290	Apricots, canned in syrup
14031	Apricots, dried
14033	Apricots, dried, stewed without sugar
14025	Apricots, raw
14036	Apricots, ready-to-eat

**12198**	Arctic roll
**13154**	Artichoke, globe, boiled, weighed as served
**13153**	Artichoke, globe, raw
**13442**	Asparagus, boiled in salted water
**13158**	Asparagus, boiled, weighed as served
**13160**	Asparagus, canned, re-heated, drained
**13157**	Asparagus, raw
**13413**	Aubergine, fried in blended oil
**13412**	Aubergine, fried in butter
**13162**	Aubergine, fried in corn oil
**13161**	Aubergine, raw
**15002**	Aubergine, stuffed with rice
**14037**	Avocado, average
**2002**	Avonmore Supermilk
**11534**	Bagels, plain
**13043**	Baked beans, canned in tomato sauce
**13046**	Baked beans, canned in tomato sauce, ?sugar & salt
**13045**	Baked beans, canned in tomato sauce, reduced sugar
**13044**	Baked beans, canned in tomato sauce, re-heated
**11283**	Bakewell tart
**17355**	Baking powder
**13163**	Bamboo shoots, canned, drained
**11573**	Banana bread
**14044**	Banana chips
**14045**	Bananas
**12394**	Banoffee pie
**17289**	Barbecue sauce
**17186**	Barley water, concentrated, made up
**11003**	Barley, pearl, boiled
**2019**	Barn Brack
**11574**	Battenburg cake
**15007**	Beanburger, red kidney bean, fried in vegetable oil
**15366**	Beanburger, soya, fried in vegetable oil
**13053**	Beansprouts, mung, boiled in salted water

**13055**	Beansprouts, mung, canned, drained
**13426**	Beansprouts, mung, raw
**13427**	Beansprouts, mung, stir-fried in blended oil
**19330**	Beef bourguignonne
**19332**	Beef casserole, made with canned cook-in sauce
**19165**	Beef chow mein, retail, reheated
**19166**	Beef curry
**19169**	Beef curry, chilled/frozen, reheated
**540**	Beef curry, retail
**19172**	Beef in sauce with vegetables, chilled/frozen, reheated
**19174**	Beef olives
**19051**	Beef pie, chilled/frozen, baked
**19076**	Beef sausages, chilled, fried
**19077**	Beef sausages, chilled, grilled
**19334**	Beef stew
**19181**	Beef Stroganoff
**19335**	Beef, stir-fried with green peppers
**19032**	Beefburgers, homemade, fried, with bun
**19034**	Beefburgers, homemade, grilled, with bun
**17506**	Beer, bitter, average
**1097**	Beer, mild, draught
**13165**	Beetroot, boiled in salted water
**13166**	Beetroot, pickled, drained
**13164**	Beetroot, raw
**15018**	Bhaji, carrot, potato and pea, with vegetable oil
**15038**	Bhaji, potato and onion
**15046**	Bhaji, vegetable, with vegetable oil
**19310**	Big Mac
**14047**	Bilberries
**17291**	Black bean sauce
**505**	Black pudding, fried
**14048**	Blackberries, raw
**14049**	Blackberries, stewed with sugar
**14050**	Blackberries, stewed without sugar

**14051**	Blackberry and apple, stewed with sugar
**17188**	Blackcurrant juice drink, conc, made up (e.g. Ribena)
**17187**	Blackcurrant juice drink, concentrated (e.g. Ribena)
**14053**	Blackcurrants, raw
**13063**	Blackeye beans, dried, boiled in unsalted water
**17101**	Boiled sweets
**19352**	Bolognese sauce with meat
**19183**	Bolognese sauce
**14807**	Bombay mix
**1034**	Bombay Mix
**17546**	Bounty bar
**1175**	Bovril
**11486**	Bran Flakes
**11005**	Bran, wheat
**11165**	Brandy snaps
**19097**	Bratwurst
**14871**	Brazil nuts
**11286**	Bread and butter pudding
**17519**	Bread sauce, made with whole milk
**11068**	Breadcrumbs, homemade
**11069**	Breadcrumbs, manufactured
**17123**	Breadsticks
**12344**	Brie
**13066**	Broad beans, boiled in unsalted water
**13069**	Broad beans, canned, re-heated, drained
**15049**	Broccoli in cheese sauce, made with whole milk
**13171**	Broccoli, green, boiled in salted water
**13172**	Broccoli, green, boiled in unsalted water
**13173**	Broccoli, green, frozen, boiled in unsalted water
**13170**	Broccoli, green, raw
**11456**	Brown bread, average
**11071**	Brown bread, large, sliced
**11073**	Brown bread, toasted
**11443**	Brown rice, boiled

**11477**	Brown rolls, crusty
**11478**	Brown rolls, soft
**1165**	Brown sauce, bottled
**13178**	Brussels sprouts, boiled in salted water
**13179**	Brussels sprouts, boiled in unsalted water
**13443**	Brussels sprouts, frozen, boiled in unsalted water
**11007**	Bulgur wheat
**13429**	Butter beans, canned, re-heated, drained
**13071**	Butter beans, dried, boiled in unsalted water
**13070**	Butter beans, dried, raw
**17485**	Butter
**17486**	Butter, spreadable
**12022**	Buttermilk
**13184**	Cabbage, boiled in salted water, average
**13444**	Cabbage, boiled in unsalted water, average
**13187**	Cabbage, Chinese, raw
**13468**	Cabbage, raw, average
**13191**	Cabbage, red, boiled in salted water
**13190**	Cabbage, red, raw
**13193**	Cabbage, Savoy, boiled in salted water
**13192**	Cabbage, Savoy, raw
**13445**	Cabbage, white, raw
**2010**	Cadbury's Crunchie
**10230**	Cake bars, chocolate, FSA Sept 2004
**10231**	Cake bars, not chocolate, FSA Sept 2004
**12345**	Camembert
**19184**	Cannelloni, chilled/frozen, reheated
**15058**	Cannelloni, spinach
**10217**	Cappuccinio/latte, FSA Sept 2004
**17357**	Carob powder
**17249**	Carrot and orange soup
**11616**	Carrot cake with topping
**13199**	Carrot juice
**13450**	Carrots, canned, re-heated, drained

**13206**	Carrots, frozen, boiled in unsalted water
**13201**	Carrots, old, boiled in salted water
**13447**	Carrots, old, boiled in unsalted water
**13446**	Carrots, old, raw
**13204**	Carrots, young, boiled in salted water
**13449**	Carrots, young, boiled in unsalted water
**13448**	Carrots, young, raw
**14811**	Cashew nuts, plain
**14812**	Cashew nuts, roasted and salted
**13210**	Cassava, boiled in unsalted water
**13213**	Cassava, gari
**13208**	Cassava, raw
**15368**	Casserole, vegetable
**15369**	Cauliflower cheese, made with semi-skimmed milk
**15064**	Cauliflower cheese, made with whole milk
**15067**	Cauliflower in white sauce, made with whole milk
**13216**	Cauliflower, boiled in salted water
**13217**	Cauliflower, boiled in unsalted water
**13218**	Cauliflower, frozen, boiled in unsalted water
**13215**	Cauliflower, raw
**13220**	Celeriac, boiled in salted water
**13219**	Celeriac, raw
**13222**	Celery, boiled in salted water
**13451**	Celery, raw
**17494**	Cereal chewy bar
**17103**	Cereal crunchy bar
**17226**	Champagne
**11458**	Chapatis, made with fat
**13224**	Chard, Swiss, boiled in unsalted water
**13223**	Chard, Swiss, raw
**11623**	Cheerios
**11550**	Cheese and onion rolls, pastry
**11219**	Cheese pastry, cooked
**12267**	Cheese pudding

**17524**	Cheese sauce packet mix, made up with s/s milk
**17523**	Cheese sauce packet mix, made up with whole milk
**17522**	Cheese sauce, made with semi-skimmed milk
**17521**	Cheese sauce, made with whole milk
**12143**	Cheese spread, flavoured
**12349**	Cheese spread, plain
**12350**	Cheese spread, reduced fat
**12131**	Cheese, Brie
**12348**	Cheese, Cheddar type, reduced fat
**12346**	Cheese, Cheddar, average
**12137**	Cheese, Cheddar, English
**12138**	Cheese, Cheddar, Irish
**12141**	Cheese, Cheddar-type, reduced fat
**12153**	Cheese, Double Gloucester
**12157**	Cheese, Feta
**12171**	Cheese, Parmesan
**12176**	Cheese, Ricotta
**12180**	Cheese, Stilton, blue
**12182**	Cheese, Wensleydale
**19314**	Cheeseburger, takeaway
**12218**	Cheesecake
**12396**	Cheesecake, fruit, individual
**11588**	Chelsea buns
**14068**	Cherries, glace
**14061**	Cherries, raw
**14065**	Cherries, stewed without sugar
**1120**	Cherry brandy
**14813**	Chestnuts
**17104**	Chew sweets
**19120**	Chick breast, marinated w garlic+herbs, chill/froz baked
**13073**	Chick pea flour
**13078**	Chick peas, canned, re-heated, drained
**13430**	Chick peas, whole, dried, boiled in unsalted water
**13074**	Chick peas, whole, dried, raw

**19054**	Chicken and mushroom pie, single crust, homemade
**19118**	Chicken breast in crumbs, chilled, fried
**19119**	Chicken breast in crumbs, chilled, grilled
**19315**	Chicken burger, takeaway
**19350**	Chicken chasseur
**19321**	Chicken chow mein, takeaway
**19322**	Chicken curry, average, takeaway
**19188**	Chicken curry, chilled/frozen, reheated
**19189**	Chicken curry, chilled/frozen, reheated, with rice
**19336**	Chicken curry, made with canned curry sauce
**549**	Chicken curry, retail
**550**	Chicken curry, with rice
**19122**	Chicken goujons, chilled/frozen, baked
**19195**	Chicken in white sauce, made with whole milk
**19123**	Chicken kiev, frozen, baked
**19197**	Chicken korma
**17254**	Chicken noodle soup, dried, as served
**19124**	Chicken nuggets, takeaway
**19055**	Chicken pie, individual, chilled/frozen, baked
**19198**	Chicken risotto
**19323**	Chicken satay
**19126**	Chicken slices
**17250**	Chicken soup, cream of, canned
**17251**	Chicken soup, cream of, canned, condensed
**17252**	Chicken soup, cream of, canned, condensed, as served
**19127**	Chicken tandoori, chilled, reheated
**19325**	Chicken tikka masala, retail
**19204**	Chicken wings, marinated, chilled/frozen, barbecued
**19205**	Chicken wings, marinated, chilled/frozen, bbq, w/w bone
**444**	Chicken, breaded, fried in vegetable oil
**19201**	Chicken, stir-fried w rice and veg, frozen, reheated
**19200**	Chicken, stir-fried with peppers in black bean sauce
**13079**	Chilli beans, canned, re-heated
**19337**	Chilli con carne

**19207**	Chilli con carne, canned
**19208**	Chilli con carne, chilled/frozen, reheated
**19209**	Chilli con carne, chilled/frozen, reheated, with rice
**17294**	Chilli sauce
**15370**	Chilli, vegetable
**10210**	Chinese cook-in-sauces, sweet & sour, FSA Sept 2004
**10211**	Chinese stir-fry sauces, FSA Sept 2004
**13024**	Chips, crinkle cut, frozen, fried in corn oil
**684**	Chips, fine cut, frozen, fried in blended oil
**13026**	Chips, fine cut, frozen, fried in corn oil
**13423**	Chips, French fries, retail
**13407**	Chips, homemade, fried in blended oil
**13021**	Chips, homemade, fried in corn oil
**13406**	Chips, homemade, fried in dripping
**13408**	Chips, homemade, fried in sunflower oil
**13410**	Chips, retail, fried in corn oil
**13411**	Chips, retail, fried in sunflower oil
**13422**	Chips, retail, fried in vegetable oil
**681**	Chips, straight cut, frozen, fried in blended oil
**13027**	Chips, thick cut, frozen, fried in corn oil
**12384**	Choc ice
**11507**	Chocolate biscuits, cream filled, full coated
**11506**	Chocolate biscuits, full coated
**11195**	Chocolate cake
**11196**	Chocolate cake, with butter icing
**11508**	Chocolate chip cookies
**17084**	Chocolate covered bar with fruit/nut wafer/biscuit
**17083**	Chocolate covered caramels
**17492**	Chocolate covered caramels
**17085**	Chocolate covered ice cream bar
**12398**	Chocolate dairy desserts
**11527**	Chocolate fudge cake
**17070**	Chocolate nut spread
**12411**	Chocolate nut sundae

**17069**	Chocolate spread
**17086**	Chocolate, cooking
**17087**	Chocolate, diabetic
**17088**	Chocolate, fancy and filled
**17089**	Chocolate, milk
**17090**	Chocolate, plain
**17491**	Chocolate, plain
**17091**	Chocolate, white
**1018**	Chocolates, fancy and filled
**19099**	Chorizo
**11290**	Christmas pudding
**17342**	Chutney, mango, oily
**17343**	Chutney, mango, sweet
**17344**	Chutney, mixed fruit
**17345**	Chutney, tomato
**17346**	Chutney, tomato, homemade
**11609**	Ciabatta
**17222**	Cider, dry
**17224**	Cider, sweet
**14291**	Clementines
**2003**	Club Orange mineral
**1080**	Coca-cola
**11488**	Coco Pops
**12082**	Cocoa powder
**12084**	Cocoa powder, made up with semi-skimmed milk
**12083**	Cocoa powder, made up with whole milk
**11197**	Coconut cake
**14819**	Coconut cream
**17105**	Coconut ice
**14820**	Coconut milk
**14873**	Coconut, desiccated
**14816**	Coconut, fresh
**16027**	Cod, coated in crumbs, frozen, fried in blended oil
**16021**	Cod, in batter, fried in blended oil

**16023**	Cod, in batter, fried in retail blend oil
**16024**	Cod, in batter, fried in sunflower oil
**16025**	Cod, in batter, frozen, baked
**17545**	Coffee and chicory essence
**17163**	Coffee and chicory essence, with water
**12026**	Coffee Compliment
**17152**	Coffee, infusion, average
**17157**	Coffee, infusion, average, with semi-skimmed milk
**17155**	Coffee, infusion, average, with single cream
**17156**	Coffee, infusion, average, with whole milk
**17153**	Coffee, infusion, strong
**17154**	Coffee, infusion, weak
**17158**	Coffee, instant
**17161**	Coffee, instant, made up with water and s/s milk
**17160**	Coffee, instant, made up with water and whole milk
**17159**	Coffee, instant, made up with water
**17164**	Coffee, Irish
**17175**	Cola
**13229**	Cole leaves, dried, boiled in unsalted water
**15077**	Coleslaw, with mayonnaise, retail
**15078**	Coleslaw, with reduced calorie dressing, retail
**68**	Common Sense Oat Bran Flakes
**12028**	Condensed milk, skimmed, sweetened
**12029**	Condensed milk, whole, sweetened
**17295**	Cook-in-sauces, canned
**17126**	Corn and starch snacks
**11490**	Corn Flakes
**17125**	Corn snacks
**19212**	Corned beef hash
**19128**	Corned beef, canned
**12386**	Cornetto type ice cream cone
**12202**	Cornetto
**11435**	Cornflour
**12351**	Cottage cheese, plain

**12352**	Cottage cheese, plain, reduced fat
**12148**	Cottage cheese, plain, with additions
**19215**	Cottage/Shepherd's pie
**19216**	Cottage/Shepherd's pie, chilled/frozen, reheated
**13231**	Courgette, boiled in unsalted water
**13415**	Courgette, fried in blended oil
**13414**	Courgette, fried in butter
**13232**	Courgette, fried in corn oil
**13230**	Courgette, raw
**11339**	Couscous
**14073**	Cranberries
**17537**	Cranberry juice
**17296**	Cranberry sauce
**12353**	Cream cheese
**11510**	Cream crackers
**11234**	Cream horns
**17242**	Cream liqueurs
**12334**	Cream, fresh, double
**12112**	Cream, fresh, half
**12332**	Cream, fresh, single
**12114**	Cream, fresh, soured
**12333**	Cream, fresh, whipping
**12121**	Cream, UHT, half
**12122**	Cream, UHT, single
**12397**	Creme caramel
**12221**	Creme caramel, homemade
**17544**	Creme egg
**17092**	Creme eggs
**12335**	Creme fraiche
**12336**	Creme fraiche, half fat
**11511**	Crispbread, rye
**11576**	Crispie cakes
**11480**	Croissants
**11293**	Crumble, apple

**11546**	Crumble, fruit
**11235**	Crumpets, fresh
**11535**	Crumpets, toasted
**11520**	Crunch biscuits, cream filled
**11491**	Crunchy Nut Corn Flakes
**13233**	Cucumber, raw
**13235**	Curly kale, boiled in salted water
**11076**	Currant bread
**11536**	Currant buns
**17297**	Curry paste
**17298**	Curry sauce, canned
**15115**	Curry, gobi aloo sag, retail
**15125**	Curry, lentil, red/masoor dahl, mung bean dahl & tomato
**15120**	Curry, lentil, red/masoor dahl, Punjabi
**16278**	Curry, prawn and mushroom
**16333**	Curry, prawn, takeaway
**15148**	Curry, spinach
**15156**	Curry, vegetable, takeaway
**11013**	Custard powder
**11537**	Custard tarts, individual
**12225**	Custard, canned
**12227**	Custard, egg
**12413**	Custard, made up with semi-skimmed milk
**12224**	Custard, made up with skimmed milk
**12412**	Custard, made up with whole milk
**12399**	Custard, ready to eat
**17017**	Dairy spread (40% fat)
**12258**	Dairy/fat spread
**11538**	Danish pastries
**14085**	Dates, dried
**14083**	Dates, raw
**11512**	Digestive biscuits, chocolate
**11513**	Digestive biscuits, plain
**17299**	Dips, sour-cream based

**15160**	Dosa, plain
**11241**	Doughnuts, custard-filled
**11242**	Doughnuts, jam
**11539**	Doughnuts, ring
**11244**	Doughnuts, ring, ice
**12228**	Dream Topping
**12229**	Dream Topping, made up with whole milk
**17300**	Dressing, blue cheese
**17301**	Dressing, 'fat free'
**17509**	Dressing, French
**17538**	Dressing, French, fat free
**17304**	Dressing, low fat
**17305**	Dressing, oil and lemon
**17306**	Dressing, thousand island
**14087**	Dried mixed fruit
**10264**	Dried pasta and sauce mix, FSA Sept 2004
**200**	Dried skimmed milk
**12030**	Dried skimmed milk
**17498**	Drinking chocolate powder
**17532**	Drinking chocolate powder, made up with s/s milk
**12096**	Drinking chocolate powder, made up with skim milk
**17533**	Drinking chocolate powder, made up with whole milk
**17499**	Drinking chocolate powder, reduced fat
**12193**	Drinking yogurt
**11603**	Dumplings
**11246**	Eclairs, fresh
**11247**	Eclairs, frozen
**12355**	Edam
**12818**	Egg fried rice
**11444**	Egg fried rice, takeaway
**10246**	Egg noodles, fine, cooked, FSA Sept 2004
**10245**	Egg noodles, medium, cooked, FSA Sept 2004
**12926**	Eggs, chicken, scrambled, with milk
**14089**	Elderberries

**11303**	Eve's pudding
**15162**	Falafel, fried in vegetable oil
**11528**	Fancy iced cakes, individual
**2004**	Fanta Orange mineral
**17027**	Fat spread (35-40% fat), polyunsaturated
**17025**	Fat spread (60% fat), with olive oil
**13242**	Fennel, Florence, boiled in salted water
**13241**	Fennel, Florence, raw
**13243**	Fenugreek leaves, raw
**12356**	Feta
**14092**	Figs, dried
**14093**	Figs, dried, stewed with sugar
**14095**	Figs, ready-to-eat
**16285**	Fish cakes, cod, homemade
**16282**	Fish cakes, fried in blended oil
**16284**	Fish cakes, fried in sunflower oil
**16281**	Fish cakes, grilled
**16291**	Fish fingers, cod, fried in sunflower oil
**16288**	Fish fingers, cod, grilled
**653**	Fish fingers, grilled
**16334**	Fish paste
**16294**	Fish pie
**11583**	Flaky pastry, cooked
**11307**	Flan case, sponge
**11305**	Flan, sponge, with fruit
**11571**	Flapjacks
**12326**	Flavoured milk, pasteurised
**12325**	Flavoured milk, pasteurised, chocolate
**17106**	Foam sweets
**19101**	Frankfurter with bun
**19100**	Frankfurter
**1157**	French dressing
**17256**	French onion soup
**10251**	Fresh egg pasta filled with cheese only, cooked, FSA

**10252**	Fresh egg pasta filled with cheese+ tomato, cooked, FSA
**10255**	Fresh egg pasta filled with meat, cooked, FSA Sept 2004
**10253**	Fresh egg pasta filled with mushrooms, cooked, FSA
**10254**	Fresh egg pasta w green veg/herbs+cheese, cooked, FS/
**10249**	Fresh egg pasta, spaghetti/tagliatelle, raw, FSA
**10250**	Fresh white egg tagliatelle, cooked, FSA Sept 2004
**12370**	Fromage frais, fruit
**17390**	Fromage frais, fruit, baby
**12369**	Fromage frais, plain
**12371**	Fromage frais, virtually fat free, natural
**11492**	Frosties
**12385**	Frozen ice cream desserts
**11529**	Fruit cake, plain, retail
**11577**	Fruit cake, rich
**11578**	Fruit cake, rich, iced
**11202**	Fruit cake, rich, retail
**116**	Fruit cake, wholemeal
**11579**	Fruit cake, wholemeal
**14096**	Fruit cocktail, canned in juice
**14097**	Fruit cocktail, canned in syrup
**17391**	Fruit desserts, baby
**17192**	Fruit drink, low calorie, concentrated, made up
**17194**	Fruit drink, low sugar, concentrated, fortified, made up
**17190**	Fruit drink/squash, concentrated, made up
**1026**	Fruit gums
**17107**	Fruit gums/jellies
**17177**	Fruit juice drink, carbonated, ready to drink
**17196**	Fruit juice drink, low calorie, ready to drink
**17195**	Fruit juice drink, ready to drink
**11493**	Fruit 'n Fibre
**17108**	Fruit pastilles
**14098**	Fruit pie filling
**11547**	Fruit pie, individual
**11596**	Fruit pie, one crust

**11597**	Fruit pie, pastry top and bottom
**11598**	Fruit pie, pastry top and bottom, blackcurrant
**14303**	Fruit salad, homemade
**17071**	Fruit spread
**17518**	Fudge
**12161**	Full fat soft cheese
**11460**	Garlic bread, pre-packed, frozen
**15179**	Garlic mushrooms
**17359**	Garlic puree
**13244**	Garlic, raw
**11205**	Gateau
**17257**	Gazpacho
**13246**	Gherkins, pickled, drained
**13245**	Gherkins, raw
**17178**	Ginger ale, dry
**13247**	Ginger root, raw
**11206**	Gingerbread
**11514**	Gingernut biscuits
**11173**	Gingernut biscuits, homemade
**12162**	Goats milk soft cheese
**12357**	Goats milk soft cheese, full fat, white rind
**12328**	Goats milk, pasteurised
**2020**	Golden Linseed
**14103**	Gooseberries, dessert, raw
**12358**	Gouda
**19340**	Goulash
**11461**	Granary bread
**11479**	Granary rolls
**14273**	Grape juice, unsweetened
**14276**	Grapefruit juice concentrate, unsweetened
**14276**	Grapefruit juice concentrate, unsweetened
**14275**	Grapefruit juice, unsweetened
**14107**	Grapefruit, canned in juice
**14108**	Grapefruit, canned in syrup

**14292**	Grapefruit, raw
**14109**	Grapes, average
**17310**	Gravy instant granules
**17311**	Gravy instant granules, made up with water
**12194**	Greek yogurt, cows
**17415**	Green beans, dried
**13082**	Green beans/French beans, boiled in salted water
**13083**	Green beans/French beans, boiled in unsalted water
**13085**	Green beans/French beans, canned, re-heated, drained
**13432**	Green beans/French beans, frozen, boiled in u/s water
**13431**	Green beans/French beans, raw
**17312**	Guacamole
**16063**	Haddock, coated in crumbs, frozen, fried in blended oil
**16062**	Haddock, coated in crumbs, frozen, raw
**16054**	Haddock, in batter, fried in retail blend oil
**16055**	Haddock, in batter, fried in sunflower oil
**16060**	Haddock, in flour, fried in sunflower oil
**17111**	Halva, semolina
**11481**	Hamburger buns
**19311**	Hamburger, takeaway
**13087**	Haricot beans, dried, boiled in unsalted water
**2023**	Hash Browns
**14874**	Hazelnuts
**17198**	High juice drink, concentrated, made up
**17313**	Hollandaise sauce, homemade
**11175**	Homemade biscuits, rubbing-in method
**17050**	Honey
**17051**	Honeycomb
**17502**	Horlicks LowFat Instant powder
**12098**	Horlicks powder, made up with whole milk
**17314**	Horseradish sauce
**13258**	Horseradish, raw
**11590**	Hot cross buns
**17315**	Hot pepper sauce

**13433**	Hummus
**12391**	Ice cream bar, chocolate coated
**17053**	Ice cream sauce, topping
**12212**	Ice cream wafers
**12205**	Ice cream, dairy, flavoured
**12392**	Ice cream, dairy, premium
**12387**	Ice cream, dairy, vanilla
**12207**	Ice cream, non-dairy, flavoured
**12208**	Ice cream, non-dairy, mixes
**12209**	Ice cream, non-dairy, reduced calorie
**12388**	Ice cream, non-dairy, vanilla
**12210**	Ice cream, with cone
**12211**	Ice cream, with wafers
**17054**	Icing, butter
**17056**	Icing, glace
**10208**	Indian cooki-in-sauces, korma/tikka masala, FSA 2004
**10209**	Indian cook-in-sauces, other, FSA, Sept 2004
**17500**	Instant drinks powder, chocolate, low calorie
**17501**	Instant drinks powder, malted
**17508**	Instant soup powder, as served
**17262**	Instant soup powder, calorie controlled, as served
**2013**	Jacobs Fig Roll
**11515**	Jaffa cakes
**10216**	Jaffa cakes, FSA Sept 2004
**11254**	Jam tarts
**11540**	Jam tarts, retail
**17072**	Jam, diabetic
**17073**	Jam, fruit with edible seeds
**17075**	Jam, reduced sugar
**17074**	Jam, stone fruit
**12237**	Jelly, made with water
**2001**	Kelloggs All Bran
**2022**	Kelloggs Country Store
**17493**	Kit Kat

**14293**	Kiwi fruit
**17212**	Lager, alcohol-free
**1099**	Lager, bottled
**19232**	Lamb rogan josh
**19234**	Lamb vindaloo
**11052**	Lasagna, boiled
**19346**	Lasagne
**19238**	Lasagne, chilled/frozen, reheated
**15187**	Lasagne, vegetable
**15189**	Lasagne, vegetable, retail
**13264**	Leeks, boiled in salted water
**13452**	Leeks, boiled in unsalted water
**13466**	Leeks, raw
**17490**	Lemon curd
**17077**	Lemon curd, homemade
**1090**	Lemon juice
**14277**	Lemon juice, fresh
**11548**	Lemon meringue pie
**16087**	Lemon sole, goujons, baked
**592**	Lemon sole, in crumbs, fried
**17179**	Lemonade
**1081**	Lemonade, bottled
**17199**	Lemonade, homemade
**14129**	Lemons, peeled
**14128**	Lemons, whole, without pips
**15197**	Lentil and potato pie
**15202**	Lentil and rice roast
**17264**	Lentil soup
**17263**	Lentil soup, canned
**13089**	Lentils, green and brown, whole, dried, raw
**13090**	Lentils, green and brown, whole, in salted water
**13434**	Lentils, red, split, dried, boiled in unsalted water
**13091**	Lentils, red, split, dried, raw
**13453**	Lettuce, average, raw

**13267**	Lettuce, butterhead, raw
**13268**	Lettuce, Cos, raw
**13269**	Lettuce, Iceberg, raw
**17200**	Lime juice cordial, concentrated
**17201**	Lime juice cordial, concentrated, made up
**1083**	Lime juice cordial, undiluted
**14279**	Lime juice, fresh
**14131**	Limes, peeled
**17112**	Liquorice allsorts
**17113**	Liquorice shapes
**14133**	Loganberries, raw
**12390**	Lollies, containing ice-cream
**12389**	Lollies, with real fruit juice
**17265**	Low calorie soup, canned
**12192**	Low calorie yogurt
**12189**	Low fat yogurt, flavoured
**12191**	Low fat yogurt, muesli/nut
**12188**	Low fat yogurt, plain
**17543**	Lucozade
**14142**	Lychees, raw
**14823**	Macadamia nuts, salted
**11562**	Macaroni cheese
**11448**	Macaroni, boiled
**10242**	Macaroni, cooked, FSA Sept 2004
**12828**	Macaroon
**11531**	Madeira cake
**17127**	Maize and rice flour snacks
**11084**	Malt bread
**11462**	Malt bread, fruited
**2009**	Maltesers
**14146**	Mandarin oranges, canned in juice
**13436**	Mange-tout peas, boiled in salted water
**13122**	Mange-tout peas, raw
**13124**	Mange-tout peas, stir-fried in blended oil

**14280**	Mango juice, canned
**13273**	Mango, unripe, raw
**14294**	Mangoes, ripe, raw
**12257**	Margarine
**17021**	Margarine, soft, polyunsaturated
**17078**	Marmalade
**17079**	Marmalade, diabetic
**1179**	Marmite
**13125**	Marrowfat peas, canned, re-heated, drained
**17547**	Mars bar
**17114**	Marshmallows
**14875**	Marzipan, retail
**17316**	Mayonnaise
**17511**	Mayonnaise, reduced calorie
**17510**	Mayonnaise, retail
**12279**	Mayonnaise, retail, reduced calorie
**2012**	McVities Hobnobs
**14153**	Melon, average
**14154**	Melon, average, weighed whole
**14295**	Melon, Canteloupe-type
**14159**	Melon, Galia
**14162**	Melon, Honeydew
**14296**	Melon, watermelon
**12829**	Meringue
**12830**	Meringue, with cream
**13028**	Microwave chips, cooked
**11085**	Milk bread
**12105**	Milk shake powder, made up with whole milk
**12327**	Milkshake, thick, takeaway
**17548**	Milky Way
**11591**	Mince pies, individual
**17267**	Minestrone soup
**17542**	Minestrone soup, canned
**17319**	Mint sauce

**17320**	Mint sauce, homemade
**13095**	Miso
**14828**	Mixed nuts and raisins
**14827**	Mixed nuts
**13283**	Mixed veg, stir-fry type, frozen, fried w blended oil
**13282**	Mixed vegetables, canned, re-heated, drained
**13281**	Mixed vegetables, frozen, boiled in salted water
**17059**	Molasses
**12400**	Mousse, chocolate
**12401**	Mousse, chocolate, low fat
**12245**	Mousse, chocolate, rich
**12402**	Mousse, fruit
**12360**	Mozzarella, fresh
**11494**	Muesli, Swiss style
**11138**	Muesli, with extra fruit
**11495**	Muesli, with no added sugar
**11260**	Muffins
**11608**	Muffins, American style, chocolate chip
**11261**	Muffins, bran
**11541**	Muffins, English style, white
**11542**	Muffins, English style, white, toasted
**17270**	Mushroom soup, cream of, canned
**13288**	Mushrooms, Chinese, dried, raw
**13285**	Mushrooms, common, boiled in salted water
**13287**	Mushrooms, common, canned, re-heated, drained
**785**	Mushrooms, common, fried in blended oil
**13416**	Mushrooms, common, fried in butter
**13286**	Mushrooms, common, fried in corn oil
**13284**	Mushrooms, common, raw
**13437**	Mushy peas, canned, re-heated
**16256**	Mussels, boiled
**16255**	Mussels, raw
**17364**	Mustard, smooth
**17365**	Mustard, wholegrain

11463	Naan bread
14297	Nectarines
13001	New potatoes, average, raw
13002	New potatoes, boiled in salted water
13003	New potatoes, boiled in unsalted water
13008	New potatoes, chipped, fried in corn oil
13006	New potatoes, frozen, `roast' in corn oil
13420	New potatoes, in skins, boiled in unsalted water
11056	Noodles, egg, boiled
11057	Noodles, fried
11059	Noodles, plain, boiled
15374	Nut roast
11612	Nutri-Grain
11517	Oat based biscuits
11489	Oat Bran Flakes, with raisins
11180	Oatcakes, homemade
11518	Oatcakes, retail
11018	Oatmeal, quick cook, raw
11017	Oatmeal, raw
13301	Okra, boiled in unsalted water
13302	Okra, stir-fried in corn oil
13010	Old potatoes, baked, flesh and skin
13011	Old potatoes, baked, flesh only
13013	Old potatoes, boiled in salted water
13014	Old potatoes, boiled in unsalted water
13421	Old potatoes, boiled in unsalted water
13402	Old potatoes, mashed with butter
13015	Old potatoes, mashed with margarine
13405	Old potatoes, mashed with polyunsatd. margarine
671	Old potatoes, roast in blended oil AND Roast potatoes
13016	Old potatoes, roast in corn oil
673	Old potatoes, roast in lard
12823	Omelette, Spanish
17525	Onion sauce, made with whole milk

**13305**	Onions, baked
**13306**	Onions, boiled in unsalted water
**794**	Onions, fried in blended oil
**13417**	Onions, fried in butter
**13307**	Onions, fried in corn oil
**797**	Onions, pickled, drained
**13309**	Onions, pickled, drained
**13304**	Onions, raw
**1084**	Orange drink, undiluted
**14284**	Orange juice concentrate, unsweetened
**14284**	Orange juice concentrate, unsweetened
**14281**	Orange juice, freshly squeezed
**14301**	Orange juice, unsweetened
**14298**	Oranges
**14176**	Oranges, weighed with peel and pips
**17129**	Oriental mix
**17504**	Ovaltine powder
**12110**	Ovaltine powder, made up with semi-skimmed milk
**12111**	Ovaltine powder, made up with skimmed milk
**12109**	Ovaltine powder, made up with whole milk
**13029**	Oven chips, frozen, baked
**13030**	Oven chips, thick cut, frozen, baked
**1182**	Oxo cubes
**17272**	Oxtail soup, canned
**17273**	Oxtail soup, dried
**17274**	Oxtail soup, dried, as served
**17321**	Oyster sauce
**16260**	Oysters, raw
**15226**	Pakora/bhajia, onion, fried in vegetable oil
**15227**	Pakora/bhajia, onion, retail
**11345**	Pakoras
**19250**	Pancakes, chicken, frozen, shallow-fried
**11346**	Pancakes, savoury
**11604**	Pancakes, savoury, made with whole milk

**11319**	Pancakes, sweet
**11320**	Pancakes, sweet, made with skimmed milk
**11601**	Pancakes, sweet, made with whole milk
**11088**	Papadums, fried
**11464**	Pappadums, takeaway
**247**	Parmesan
**12361**	Parmesan, fresh
**13313**	Parsnip, boiled in salted water
**13314**	Parsnip, boiled in unsalted water
**13454**	Parsnip, boiled in unsalted water
**13312**	Parsnip, raw
**14285**	Passion fruit juice
**14178**	Passion fruit
**10265**	Pasta and sauce mix, cooked, FSA - NO DATA
**10241**	Pasta bows, cooked, FSA Sept 2004
**10263**	Pasta sauce, carbonara type, FSA Sept 2004
**17323**	Pasta sauce, tomato based
**17322**	Pasta sauce, white
**10240**	Pasta shells, cooked, FSA Sept 2004
**10238**	Pasta twists, cooked in salted water, FSA Sept 2004
**10239**	Pasta twists, cooked in salted water, FSA Sept 2004
**10237**	Pasta twists, cooked, FSA Sept 2004
**19347**	Pasta with meat and tomato sauce
**11451**	Pasta, fresh, cheese and vegetable stuffed, cooked
**11450**	Pasta, plain, fresh, cooked
**19317**	Pate, liver
**19145**	Pate, meat, low fat
**12403**	Pavlova, no fruit
**12404**	Pavlova, with fruit and cream
**14180**	Paw-paw, raw
**14188**	Peaches, canned in juice
**14185**	Peaches, dried
**14299**	Peaches, raw
**14876**	Peanut butter, smooth

**14830**	Peanut butter, wholegrain
**14882**	Peanuts and raisins
**14882**	Peanuts and raisins
**14878**	Peanuts, dry roasted
**14877**	Peanuts, plain
**14835**	Peanuts, raisins and chocolate chips
**14834**	Peanuts, roasted and salted
**14190**	Pears, average, raw
**14192**	Pears, average, raw, peeled
**14195**	Pears, average, stewed without sugar
**14197**	Pears, canned in juice
**14201**	Pears, Conference, raw
**13128**	Peas, boiled in salted water
**13439**	Peas, boiled in unsalted water
**13441**	Peas, canned, re-heated, drained
**13131**	Peas, dried, boiled in unsalted water
**13465**	Peas, frozen, boiled in salted water
**13440**	Peas, frozen, boiled in unsalted water
**13132**	Peas, frozen, raw
**14837**	Pecan nuts
**19108**	Pepperami
**17117**	Peppermints
**13316**	Peppers, capsicum, chilli, green, raw
**13317**	Peppers, capsicum, chilli, red, raw
**13319**	Peppers, capsicum, green, boiled in salted water
**13318**	Peppers, capsicum, green, raw
**13321**	Peppers, capsicum, red, boiled in salted water
**13320**	Peppers, capsicum, red, raw
**13322**	Peppers, capsicum, yellow, raw
**15238**	Peppers, stuffed with rice
**15240**	Pesto sauce
**10260**	Pesto, green, FSA Sept 2004
**10261**	Pesto, red, FSA Sept 3004
**13138**	Petit pois, frozen, boiled in unsalted water

**17349**	Pickle, lime, oily
**17351**	Pickle, mixed vegetables
**17352**	Pickle, sweet
**15241**	Pie, Quorn and vegetable
**15379**	Pie, vegetable
**15245**	Pilaf, rice with spinach
**11348**	Pilau rice
**14839**	Pine nuts
**14287**	Pineapple juice concentrate, unsweetened
**14287**	Pineapple juice concentrate, unsweetened
**14286**	Pineapple juice, unsweetened
**14211**	Pineapple, canned in juice
**14212**	Pineapple, canned in syrup
**14210**	Pineapple, dried
**14208**	Pineapple, raw
**13107**	Pinto beans, dried, boiled in unsalted water
**13106**	Pinto beans, dried, raw
**14840**	Pistachio nuts, roasted and salted
**11465**	Pitta bread, white
**11552**	Pizza base, raw
**11349**	Pizza
**15252**	Pizza, cheese and tomato
**11613**	Pizza, cheese and tomato, deep pan
**11553**	Pizza, cheese and tomato, frozen
**15254**	Pizza, cheese and tomato, retail, frozen
**11614**	Pizza, cheese and tomato, thin base
**11559**	Pizza, chicken topped, chilled
**11560**	Pizza, fish topped, takeaway
**11558**	Pizza, ham and pineapple, chilled
**11556**	Pizza, meat topped
**11557**	Pizza, vegetarian
**16112**	Plaice, in batter, fried in retail blend oil
**16113**	Plaice, in batter, fried in sunflower oil
**13324**	Plantain, boiled in unsalted water

**14300**	Plums, average, raw
**14215**	Plums, average, stewed with sugar
**14288**	Pomegranate juice, fresh
**17130**	Popcorn, candied
**17131**	Popcorn, plain
**19254**	Pork and beef meatballs in tomato sauce
**19090**	Pork and beef, economy sausages, chilled, raw
**19348**	Pork casserole, made with canned cook-in sauce
**19257**	Pork chops in mustard and cream
**19079**	Pork sausages, chilled, fried
**19080**	Pork sausages, chilled, grilled
**19082**	Pork sausages, frozen, fried
**19083**	Pork sausages, frozen, grilled
**19086**	Pork sausages, reduced fat, chilled/frozen, grilled
**19260**	Pork spare ribs, 'barbecue style'
**19262**	Pork spare ribs, 'barbecue style', chilled/frozen, reheated
**19261**	Pork spare ribs, 'barbecue style', homemade, w/w bone
**19263**	Pork spare ribs, 'bbq style', chill/froz, reheated, w bone
**11142**	Porridge, made with milk and water
**11143**	Porridge, made with water
**11569**	Porridge, made with water
**11570**	Porridge, made with whole milk
**17276**	Potato and leek soup
**15257**	Potato cakes, fried in lard
**15258**	Potato cakes, fried in vegetable oil
**17495**	Potato crisps
**17134**	Potato crisps, crinkle cut
**17135**	Potato crisps, jacket
**17496**	Potato crisps, low fat
**17139**	Potato crisps, thick, crinkle-cut
**17138**	Potato crisps, thick-cut
**13038**	Potato croquettes, fried in blended oil
**13039**	Potato flour
**17142**	Potato rings

**13040**	Potato waffles, frozen, cooked
**10228**	Potato wedges, oven baked, FSA Sept 2004
**15261**	Potatoes with eggs
**15260**	Potatoes, duchesse
**11551**	Prawn crackers, takeaway
**10223**	Premium chilled carton soup, carrot+coriander, FSA
**10224**	Premium chilled carton soup, mushroom, FSA Sept 2004
**10225**	Premium chilled carton soup, tomato, FSA Sept 2004
**19095**	Premium sausages, chilled, grilled
**17145**	Pretzels
**12362**	Processed cheese, plain
**12363**	Processed cheese, slices, reduced fat
**13140**	Processed peas, canned, re-heated, drained
**12405**	Profiteroles with sauce
**32047**	Protifar/100g (NC-000048)
**14289**	Prune juice
**14231**	Prunes
**14237**	Prunes, canned in juice
**14239**	Prunes, ready-to-eat
**11224**	Puff pastry, frozen, raw
**17147**	Puffed potato products
**11144**	Puffed Wheat
**14842**	Pumpkin seeds
**13327**	Pumpkin, boiled in salted water
**2021**	Quaker Snack A Jack Rice Cakes
**19312**	Quarterpounder with cheese, takeaway
**19048**	Quarterpounder, takeaway
**11323**	Queen of puddings
**12923**	Quiche, cheese and egg
**12925**	Quiche, Lorraine
**10205**	Quiche, meat, retail, FSA Sept 2004
**10207**	Quiche, vegetable, retail, FSA Sept 2004
**14843**	Quinoa
**17366**	Quorn, myco-protein

**13455**	Quorn, pieces, as purchased
**13330**	Radish, red, raw
**14242**	Raisins
**17324**	Raita
**14247**	Raspberries, frozen
**14244**	Raspberries, raw
**14245**	Raspberries, stewed with sugar
**14246**	Raspberries, stewed without sugar
**15263**	Ratatouille
**15264**	Ratatouille, retail
**11621**	Ravioli, canned in tomato sauce
**11496**	Ready Brek
**13435**	Red kidney beans, canned, re-heated, drained
**13110**	Red kidney beans, dried, boiled in unsalted water
**13109**	Red kidney beans, dried, raw
**11038**	Red rice, boiled
**17228**	Red wine
**14249**	Redcurrants, raw
**11617**	Reduced fat cake
**15265**	Re-fried beans
**17354**	Relish, burger/chilli/tomato
**17353**	Relish, corn/cucumber/onion
**14254**	Rhubarb, stewed without sugar
**1085**	Ribena, undiluted
**11618**	Rice cakes
**10222**	Rice cakes, FSA Sept 2004
**11497**	Rice Krispies
**2007**	Rice Milk (Rice Dream Original with added Calcium)
**10248**	Rice noodles fine, cooked, FSA Sept 2004
**12406**	Rice pudding, canned
**12407**	Rice pudding, canned, low fat
**2011**	Rich Tea biscuits (Dunnes Stores)
**11498**	Ricicles
**11605**	Risotto, plain

**15378**	Risotto, vegetable
**11210**	Rock cakes
**16141**	Rock Salmon, in batter, fried in sunflower oil w/bones
**17181**	Root beer
**17229**	Rose wine, medium
**13114**	Runner beans, boiled in unsalted water
**11161**	Rusks, plain
**11091**	Rye bread
**17512**	Salad cream
**17327**	Salad cream, reduced calorie
**15286**	Salad, bean, retail
**15287**	Salad, beetroot
**15288**	Salad, carrot and nut with French dressing, retail
**15380**	Salad, green
**15293**	Salad, pasta
**15294**	Salad, pasta, wholemeal
**15295**	Salad, potato, with French dressing
**15296**	Salad, potato, with mayonnaise
**15297**	Salad, potato, with mayonnaise, retail
**15298**	Salad, potato, with reduced calorie dressing, retail
**15381**	Salad, rice
**15300**	Salad, rice, brown
**19110**	Salami
**13334**	Salsify, raw
**11353**	Samosas, meat
**11354**	Samosas, vegetable
**15305**	Samosas, vegetable, retail
**11182**	Sandwich biscuits
**11519**	Sandwich biscuits, cream filled
**11516**	Sandwich biscuits, jam filled
**17328**	Sandwich spread
**11564**	Sandwich, Cheddar cheese and pickle, white bread
**11565**	Sandwich, Chicken salad, white bread
**11567**	Sandwich, Egg mayonnaise, white bread

**11568**	Sandwich, Tuna mayonnaise, white bread
**16217**	Sardines, canned in tomato sauce
**14257**	Satsumas
**15306**	Sauce, curry, onion, with butter
**15307**	Sauce, curry, onion, with vegetable oil
**15308**	Sauce, curry, sweet
**15309**	Sauce, curry, tomato and onion
**17332**	Sauce, dry mix, made up
**17329**	Sauce, dry, casserole mix
**17330**	Sauce, dry, casserole mix, made up
**15311**	Sauce, tomato and mushroom
**15310**	Sauce, tomato base
**17333**	Sauce, tomato base, homemade
**13336**	Sauerkraut
**522**	Sausage roll, flaky pastry
**523**	Sausage roll, short pastry
**19067**	Sausage rolls, flaky pastry, homemade
**19066**	Sausage rolls, puff pastry
**11620**	Savoury rice, cooked
**16262**	Scallops, steamed
**16244**	Scampi, in breadcrumbs, frozen, fried in sunflower oil
**11264**	Scones, cheese
**11265**	Scones, fruit
**11543**	Scones, fruit, retail
**11592**	Scones, plain
**11593**	Scones, wholemeal
**17277**	Scotch broth
**11544**	Scotch pancakes, retail
**16306**	Seafood cocktail
**16305**	Seafood pasta, retail
**12312**	Semi-skimmed milk, average
**186**	Semi-skimmed milk, pasteurised
**12313**	Semi-skimmed milk, pasteurised
**12009**	Semi-skimmed milk, pasteurised, average

**12010**	Semi-skimmed milk, pasteurised, fortified plus SMP
**12418**	Semi-skimmed milk, pasteurised, summer
**12419**	Semi-skimmed milk, pasteurised, winter
**11521**	Semi-sweet biscuits
**11024**	Semolina, raw
**14844**	Sesame seeds
**13342**	Shallots, raw
**17217**	Shandy
**14259**	Sharon fruit
**19270**	Shepherd's pie
**17119**	Sherbert sweets
**17235**	Sherry, dry
**11523**	Shortbread
**11585**	Shortcrust pastry, cooked
**11522**	Short-sweet biscuits
**11499**	Shredded Wheat
**11500**	Shreddies
**12306**	Skimmed milk, average
**12307**	Skimmed milk, pasteurised
**12003**	Skimmed milk, pasteurised, fortified plus SMP
**12311**	Skimmed milk, sterilised
**12006**	Skimmed milk, UHT, fortified plus SMP
**17096**	Smartie-type sweets
**17549**	Snickers
**11092**	Soda bread
**17182**	Soda, club
**12393**	Sorbet, fruit
**12826**	Souffle, plain
**17334**	Soy sauce
**13116**	Soya beans, dried, boiled in unsalted water
**12043**	Soya milk, flavoured
**12042**	Soya milk, plain
**15314**	Soya mince, granules
**12381**	Soya, alternative to yogurt, fruit

**12330**	Soya, non-dairy altern to milk, sweetened, Ca enriched
**12331**	Soya, non-dairy alternative to milk, unsweetened
**19353**	Spaghetti bol, chilled/frozen, reheated, w spaghetti
**19272**	Spaghetti bolognese
**11357**	Spaghetti, canned in tomato sauce
**10266**	Spaghetti, canned in tomato sauce, FSA Sept 2004
**10243**	Spaghetti, cooked, FSA Sept 2004
**11453**	Spaghetti, white, boiled
**11455**	Spaghetti, wholemeal, boiled
**11501**	Special K
**13344**	Spinach, boiled in salted water
**13457**	Spinach, boiled in unsalted water
**17417**	Spinach, dried
**13458**	Spinach, frozen, boiled in unsalted water
**13456**	Spinach, raw
**11580**	Sponge cake
**11581**	Sponge cake, fatless
**11213**	Sponge cake, jam filled
**11214**	Sponge cake, with butter icing
**11532**	Sponge cake, with dairy cream and jam
**11325**	Sponge pudding
**11326**	Sponge pudding, with dried fruit
**12364**	Spreadable cheese, soft white, full fat
**12366**	Spreadable cheese, soft white, low fat
**13350**	Spring greens, boiled in unsalted water
**13348**	Spring greens, raw
**13352**	Spring onions, bulbs and tops, raw
**13351**	Spring onions, bulbs only, raw
**19327**	Spring rolls, meat, takeaway
**13356**	Squash, butternut, baked
**13355**	Squash, butternut, raw
**16267**	Squid, dried
**16266**	Squid, in batter, fried in sunflower oil
**16263**	Squid, raw

**11151**	Start
**19071**	Steak and kidney pie, double crust, homemade
**535**	Steak and kidney pie, pastry top only
**19072**	Steak and kidney pudding, canned
**19069**	Steak and kidney/beef pie, individual, chill/frozen, baked
**17219**	Stout, Guinness
**14262**	Strawberries, canned in syrup
**14261**	Strawberries, frozen
**14260**	Strawberries, raw
**11272**	Strawberry tartlets
**17371**	Stuffing mix
**17372**	Stuffing mix, made up with water
**11606**	Stuffing, sage and onion
**17373**	Stuffing, sage and onion, homemade
**11503**	Sugar Puffs
**17060**	Sugar, brown
**17061**	Sugar, Demerara
**17062**	Sugar, icing
**17063**	Sugar, white
**13144**	Sugar-snap peas, boiled in salted water
**13143**	Sugar-snap peas, raw
**11504**	Sultana Bran
**14263**	Sultanas
**14845**	Sunflower seeds
**14846**	Sunflower seeds, toasted
**10219**	Sushi, tuna nigiri, FSA Sept 20
**10220**	Sushi, vegetable, FSA Sept 2004
**13361**	Swede, boiled in unsalted water
**13359**	Swede, raw
**19324**	Sweet and sour chicken, takeaway
**19349**	Sweet and sour pork
**17335**	Sweet and sour sauce, canned
**17336**	Sweet and sour sauce, take-away
**13363**	Sweet potato, baked

**13464**	Sweet potato, boiled in salted water
**13463**	Sweet potato, raw
**13367**	Sweetcorn, baby, canned, drained
**13366**	Sweetcorn, baby, fresh and frozen, boiled in salt water
**13369**	Sweetcorn, kernels, boiled in salted water
**13370**	Sweetcorn, kernels, boiled in unsalted water
**13459**	Sweetcorn, kernels, canned, re-heated, drained
**13368**	Sweetcorn, kernels, raw
**13373**	Sweetcorn, on-the-cob, whole, boiled in salted water
**13374**	Sweetcorn, on-the-cob, whole, in unsalted water
**11216**	Swiss roll
**11533**	Swiss roll, chocolate, individual
**11217**	Swiss rolls, chocolate, individual
**17065**	Syrup, golden
**17066**	Syrup, golden, pouring
**17067**	Syrup, maple
**16335**	Szechuan prawns with vegetables, takeaway
**14847**	Tahini paste
**14266**	Tangerines
**11027**	Tapioca, raw
**17337**	Tartare sauce
**17172**	Tea, herbal, infusion
**17169**	Tea, infusion, average, with semi-skimmed milk
**17168**	Tea, infusion, average, with whole milk
**17173**	Tea, lemon, instant powder
**17174**	Tea, lemon, instant powder, with water
**11273**	Teacakes, fresh
**11545**	Teacakes, toasted
**12343**	Tip Top dessert topping
**17120**	Toffees, mixed
**15320**	Tofu spread
**13119**	Tofu, soya bean, steamed
**13120**	Tofu, soya bean, steamed, fried
**1163**	Tomato chutney

**13382**	Tomato juice
**17513**	Tomato ketchup
**17516**	Tomato puree
**1173**	Tomato sauce
**17278**	Tomato soup, cream of, canned
**17280**	Tomato soup, cream of, canned, condensed, as served
**17281**	Tomato soup, dried
**17282**	Tomato soup, dried, as served
**13461**	Tomatoes, canned, whole contents
**13388**	Tomatoes, cherry, raw
**828**	Tomatoes, fried in blended oil
**13385**	Tomatoes, fried in corn oil
**13419**	Tomatoes, fried in lard
**13467**	Tomatoes, grilled
**13460**	Tomatoes, raw
**17375**	Tomatoes, sun dried
**17184**	Tonic water
**17238**	Tonic wine
**17497**	Tortilla chips
**11093**	Tortillas, made with wheat flou
**10212**	Traditional cook-in-sauces, tomato based, FSA
**10213**	Traditional cook-in-sauces, white sauce based, FSA 2004
**17068**	Treacle, black
**12417**	Trifle
**12409**	Trifle, fruit
**2008**	Tropicana Multivitamins Fruit Juice
**17098**	Truffles, mocha
**19283**	Turkey and pasta bake
**19155**	Turkey roast, frozen, cooked
**19157**	Turkey slices
**17121**	Turkish delight, with nuts
**17122**	Turkish delight, without nuts
**13390**	Turnip, boiled in salted water
**13391**	Turnip, boiled in unsalted water

**13389**	Turnip, raw
**17550**	Twix
**11275**	Vanilla slices
**15326**	Vegebanger mix, made with water, fried in vegetable oil
**15340**	Vegeburger mix, made up with water and egg, grilled
**15333**	Vegeburger mix, made up with water
**15335**	Vegeburger mix, made up with water, fried in veg oil
**15338**	Vegeburger mix, made w water+egg, fried in sunflow oil
**15330**	Vegeburger, retail, fried in vegetable oil
**15331**	Vegeburger, retail, grilled
**15382**	Vegetable bake
**15362**	Vegetable kiev, baked
**17376**	Vegetable puree
**17283**	Vegetable soup
**17284**	Vegetable soup, canned
**17286**	Vegetable soup, dried, as served
**15345**	Vegetable stir fry mix, fried in sunflower oil
**15346**	Vegetable stir fry mix, fried in vegetable oil
**15364**	Vegetables, stir-fried, takeaway
**15365**	Vegetarian sausages, baked/grilled
**15347**	Vine leaves, stuffed with rice
**17339**	Vinegar
**11524**	Wafer biscuits, filled
**11509**	Wafers, filled, chocolate, full coated
**11276**	Waffles
**14879**	Walnuts
**11187**	Water biscuits
**13395**	Water chestnuts, canned, drained
**13394**	Water chestnuts, raw
**13462**	Watercress, raw
**11505**	Weetabix
**11156**	Weetaflake 'n' raisin
**11155**	Weetaflake
**11157**	Weetos

**17151**	Wheat crunchies
**11439**	Wheat flour, white, plain
**11440**	Wheat flour, white, self-raising
**11441**	Wheat flour, wholemeal
**11467**	Wheatgerm bread
**11098**	Wheatgerm bread, average
**11622**	Wheatgerm
**11099**	White bread, average
**11470**	White bread, farmhouse or split tin, freshly baked
**11471**	White bread, French stick
**50**	White bread, fried in blended oil
**11469**	White bread, fried in lard
**11105**	White bread, fried
**11100**	White bread, large, crusty
**11108**	White bread, Scottish batch, unwrapped
**11109**	White bread, Scottish batch, wrapped
**11468**	White bread, sliced
**11475**	White bread, toasted
**11110**	White bread, Vienna
**11472**	White bread, 'with added fibre'
**11473**	White bread, 'with added fibre', toasted
**12368**	White cheese, average
**11041**	White rice, basmati, raw
**11446**	White rice, easy cook, boiled
**11610**	White rice, fried
**11047**	White rice, glutinous, boiled
**11050**	White rice, polished, boiled
**11482**	White rolls, crusty
**11483**	White rolls, soft
**12304**	White sauce packet mix, made up with s/s milk
**12303**	White sauce packet mix, made up with whole milk
**17528**	White sauce, savoury, made with semi-skimmed milk
**17527**	White sauce, savoury, made with whole milk
**17529**	White sauce, sweet, made with whole milk

**17230**	White wine, dry
**17231**	White wine, medium
**17232**	White wine, sparkling
**16162**	Whiting, in crumbs, fried in blended oil
**16166**	Whiting, in crumbs, fried in sunflower oil
**12185**	Whole milk yogurt, fruit
**12375**	Whole milk yogurt, fruit
**12187**	Whole milk yogurt, goats
**12186**	Whole milk yogurt, 'organic'
**12184**	Whole milk yogurt, plain AND greek yoghurt
**12315**	Whole milk, average
**12316**	Whole milk, pasteurised
**12317**	Whole milk, pasteurised, summer
**12318**	Whole milk, pasteurised, winter
**12320**	Whole milk, UHT
**11476**	Wholemeal bread, average
**11114**	Wholemeal bread, large
**11115**	Wholemeal bread, small, sliced
**11116**	Wholemeal bread, small, unsliced
**11611**	Wholemeal bread, toasted
**11572**	Wholemeal crackers
**11484**	Wholemeal rolls
**19313**	Whopper burger
**17340**	Worcestershire sauce
**13400**	Yam, boiled in unsalted water
**17517**	Yeast extract (e.g. Marmite)
**17379**	Yeast, dried
**17408**	Yogurt, fruit, baby
**12377**	Yogurt, greek style, fruit
**12376**	Yogurt, greek style, plain
**12380**	Yogurt, low fat, fruit
**12379**	Yogurt, low fat, plain
**12374**	Yogurt, twinpot, thick and creamy with fruit
**12382**	Yogurt, virtually fat free/diet, fruit

**12383**	Yogurt, virtually fat free/diet, plain
**11607**	Yorkshire pudding
